# Design Considerations for Macroencapsulation Devices for Stem Cell Derived Islets for the Treatment of Type 1 Diabetes

**DOI:** 10.1002/advs.202100820

**Published:** 2021-06-21

**Authors:** Debkalpa Goswami, Daniel A. Domingo‐Lopez, Niamh A. Ward, Jeffrey R. Millman, Garry P. Duffy, Eimear B. Dolan, Ellen T. Roche

**Affiliations:** ^1^ Institute for Medical Engineering and Science Massachusetts Institute of Technology Cambridge MA 02139 USA; ^2^ Department of Anatomy College of Medicine, Nursing, and Health Sciences National University of Ireland Galway Galway H91 TK33 Ireland; ^3^ Department of Biomedical Engineering School of Engineering College of Science and Engineering National University of Ireland Galway Galway H91 TK33 Ireland; ^4^ Division of Endocrinology, Metabolism & Lipid Research Washington University School of Medicine St. Louis MO 63110 USA; ^5^ Department of Biomedical Engineering Washington University in St. Louis St. Louis MO 63110 USA; ^6^ Advanced Materials and BioEngineering Research Centre (AMBER) Trinity College Dublin Dublin D02 PN40 Ireland; ^7^ CÚRAM, Centre for Research in Medical Devices National University of Ireland Galway Galway H91 TK33 Ireland; ^8^ Department of Mechanical Engineering Massachusetts Institute of Technology Cambridge MA 02139 USA

**Keywords:** implantable therapeutic devices, macroencapsulation devices, stem cell derived islets, type 1 diabetes

## Abstract

Stem cell derived insulin producing cells or islets have shown promise in reversing Type 1 Diabetes (T1D), yet successful transplantation currently necessitates long‐term modulation with immunosuppressant drugs. An alternative approach to avoiding this immune response is to utilize an islet macroencapsulation device, where islets are incorporated into a selectively permeable membrane that can protect the transplanted cells from acute host response, whilst enabling delivery of insulin. These macroencapsulation systems have to meet a number of stringent and challenging design criteria in order to achieve the ultimate goal of reversing T1D. In this progress report, the design considerations and functional requirements of macroencapsulation systems are reviewed, specifically for stem‐cell derived islets (SC‐islets), highlighting distinct design parameters. Additionally, a perspective on the future for macroencapsulation systems is given, and how incorporating continuous sensing and closed‐loop feedback can be transformative in advancing toward an autonomous biohybrid artificial pancreas.

## Graphical abstract

Transplantation of stem‐cell‐derived islets (SC‐islets) is a promising treatment option for the reversal of Type 1 diabetes. This progress report reviews the design considerations and functional requirements for macroencapsulation systems, specifically for SC‐islets, highlighting distinct design parameters. Incorporating continuous sensing and closed‐loop feedback in future macroencapsulation systems could be transformative in advancing toward an autonomous biohybrid artificial pancreas.

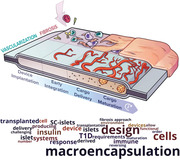

## 1 Introduction

Transplantation of stem cell derived insulin producing cells is a promising treatment option for the reversal of Type 1 Diabetes (T1D).^[^ ^1^, ^2^, ^3^ ^]^ However, successful islet transplantation remains challenging due to the host immune response, and long‐term modulation of this response with immunosuppressant drugs is undesirable due to a multitude of negative side effects.^[^ ^4^, ^5^ ^]^ The most established method of islet transplantation, known as the Edmonton protocol, involves infusion of cadaveric islets into the liver via the portal vein alongside an immunosuppressive regime to prevent immune rejection of the transplanted cells. Although allogeneic or xenogeneic islet transplantation has had some success, there are associated limitations including graft loss, rejection, and death during infusion,^[^ ^6^, ^7^, ^8^, ^9^ ^]^ with 50–70% of transplanted cells estimated to be lost prior to engraftment in the liver.^[^ ^10^, ^11^ ^]^ In terms of islet numbers, 10 000 islet equivalent (IEQ) per kg is recommended for intra‐portal transplantation via the Edmonton protocol; however, only an estimated 3000–5000 IEQ per kg remain viable following transplantation.^[^ ^12^ ^]^ To date, limited availability of functional donor pancreases has prevented widespread implementation of islet transplantation as a treatment for T1D, since two or more donor pancreases are often needed to achieve enough islets to reverse diabetes (10 000 IEQ per kg).

**Figure 1:**
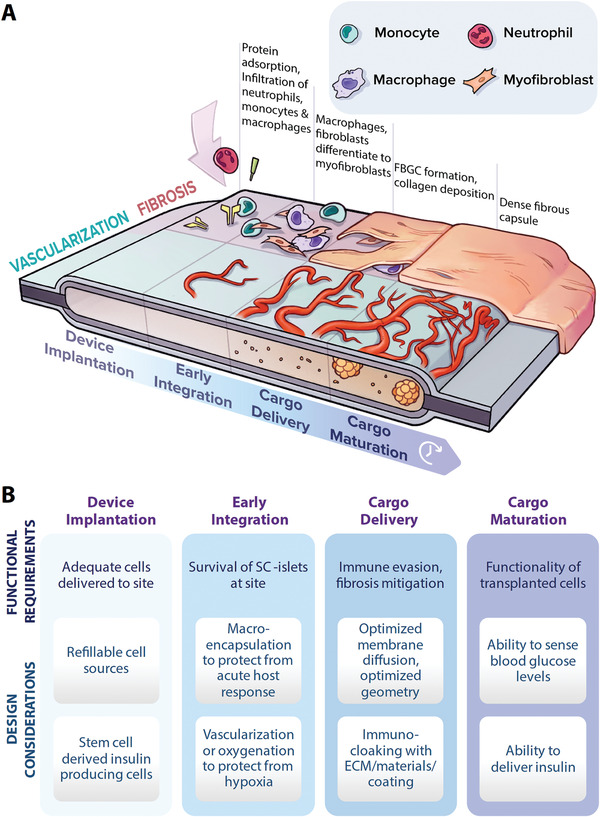
Design considerations for macroencapsulation devices. A) Graphical representation of the temporally dynamic therapeutic cell cargo and peri‐implant tissue during device implantation, device integration, cargo delivery, and cargo maturation—depicting how the processes of fibrosis (negatively affecting device function) and vascularization (positively affecting device function) are both increasing with time. B) Pertinent functional requirements and design considerations at each of the stages.

A macroencapsulation device incorporates islets into a selectively permeable membrane which evades the immune response, whilst enabling delivery of insulin from the transplanted cells.^[^ ^13^, ^14^, ^15^ ^]^ These macroencapsulation systems have to meet a number of stringent and challenging functional requirements in order to achieve the ultimate goal of reversing T1D. Critically, they need to maintain viability of the transplanted cells, and this is further complicated by the fact that stem cell derived insulin producing cells have been shown to exhibit major changes and phenotypic maturation when transplanted in vivo.^[^ ^16^ ^]^ In addition to a maturing cell cargo, the peri‐implant environment is temporally dynamic, as the processes of implant vascularization and fibrosis evolve from device implantation to early integration in parallel to maturation of the transplanted cells (**Figure** **1**).^[^ ^17^ ^]^

Advances in islet macroencapsulation systems lay an excellent foundation for the development of devices with bespoke design for stem‐cell derived insulin producing cells. Without combination with an immune protection approach, such as immunosuppressants or encapsulation devices, SC‐islets could not treat diabetes caused by autoimmune destruction of the insulin‐producing cells, such as in T1D. Some of the major issues faced by xenogeneic islet macroencapsulation, for example, immune response, fibrosis, and hypoxia, will also be relevant for devices that have specific design elements for SC‐islets.

## 2 Stem Cell Derived Islets

**Figure 2:**
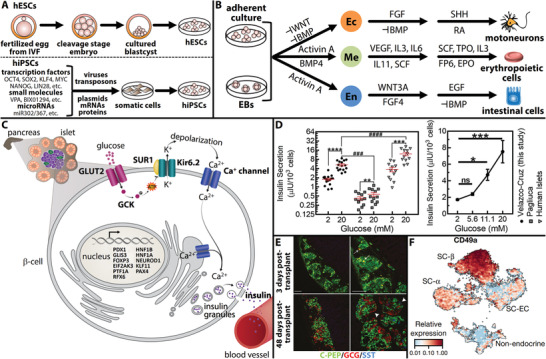
SC‐islets for diabetes cell replacement therapy. A) Derivation of human embryonic stem cells (hESCs) and human induced pluripotent stem cells (hiPSCs). Adapted with permission.^[^ ^60^ ^]^ Copyright 2013, Company of Biologists. B) In vitro differentiation of hPSCs is possible either in adherent culture or in suspension culture via embryoid body (EB) formation. Adapted with permission.^[^ ^60^ ^]^ Copyright 2013, Company of Biologists. C) Response mechanism of functional *β*‐cells to increasing glucose levels involves increasing insulin secretion. Adapted with permission.^[^ ^20^ ^]^ Copyright 2013, Company of Biologists. D) Improved insulin secretion via a differentiation strategy focused on modulating transforming growth factor *β* (TGF‐*β*) signaling and controlling three‐dimensional cellular cluster assembly. Adapted with permission.^[^ ^2^ ^]^ Copyright 2019, Elsevier. E) Enriched *β*‐clusters achieve in vivo functionality within 3 days after transplant and retain function long‐term (≈48 days). Scale bars are 100 µm. Adapted with permission.^[^ ^33^ ^]^ Copyright 2019, Springer Nature. F) Purification of SC‐*β*‐cells using CD49a as a surface marker of the *β*‐cell population, allowing magnetic sorting to a purity of 80%. Adapted with permission.^[^ ^38^ ^]^ Copyright 2019, Springer Nature.

Human pluripotent stem cells (hPSCs), including embryonic and induced pluripotent stem cells, can be expanded indefinitely in culture and subsequently induced to differentiate into any cell type found in the body (**Figure** **2**A,B).^[^ ^18^, ^19^ ^]^ These capabilities have motivated much interest into these cells as a potential renewable source of replacement hPSC‐derived islets (SC‐islets) for diabetes cell replacement therapy (Figure 2C).^[^ ^20^ ^]^ Early work focused on the generation of key intermediate cell types that are first required before SC‐islets can be produced in vitro. To produce SC‐islets, hPSCs must first differentiate to definitive endoderm^[^ ^21^ ^]^ and pancreatic progenitors expressing the transcription factors PDX1+.^[^ ^22^ ^]^ These PDX1+ pancreatic progenitors could spontaneously differentiate to SC‐islets after transplantation into mice.^[^ ^23^, ^24^, ^25^ ^]^ Subsequent studies further refined and expanded the understanding of the differentiation protocols for making these progenitors and immature non‐functional endocrine cells.^[^ ^26^, ^27^, ^28^, ^29^ ^]^

These early studies laid the foundation for the development of strategies focused on the differentiation of hPSCs to SC‐islets in vitro in a stage‐specific fashion through these developmental intermediates, attempting to recapitulate embryonic events or cues.^[^ ^3^, ^30^ ^]^ SC‐islets from these first studies produced pancreatic endocrine hormones and contained *β*‐like cells, which co‐expressed defining *β* cells markers, such as insulin and NKX6‐1. SC‐islets could secrete insulin in response to glucose and prevent diabetes once transplanted into mice. However, these cells lacked or had lower expression of markers defining mature adult human islets, secreted less insulin per cell, and lacked proper kinetic release of insulin. The immaturity of SC‐islets led to subsequent studies to define the proper conditions to produce mature SC‐islets, including investigating media composition for differentiation and maturation,^[^ ^2^, ^31^, ^32^ ^]^ controlling of three‐dimensional cellular cluster assembly,^[^ ^2^, ^33^ ^]^ and targeting the state of the actin cytoskeleton.^[^ ^34^ ^]^ These advances have resulted in improved SC‐islets, particularly in terms of insulin secretion in vitro and in vivo, approaching that of primary human islets (Figure 2D).

The promise of SC‐islets in diabetes cell replacement therapy has prompted investigation of different approaches to better facilitate clinical delivery of these cells into patients. Using cell sorting to improve the purity of cellular preparations has recently received significant attention. Nair et al. used a genetically encoded GFP reporter of *INS* gene activity to purify cells with active insulin gene transcription to enrich the proportion of hPSC‐derived *β*‐like cells (Figure 2E).^[^ ^33^ ^]^ Other sorting approaches have focused on surface markers that could be extended to non‐genetically engineered cell lines to both enrich progenitors^[^ ^35^, ^36^, ^37^ ^]^ and hPSC‐derived *β*‐like cells (Figure 2F).^[^ ^38^, ^39^ ^]^

SC‐islets and other stem cell products have been combined with other islet non‐endocrine cell types. In particular, this has been done with endothelial cells that, while normally present in primary islets,^[^ ^20^ ^]^ are not produced with the differentiation protocols that generate SC‐islets.^[^ ^40^, ^41^, ^42^, ^43^ ^]^ However, while the theoretical inclusion of endothelial cells should add noticeable improvements in SC‐islets, to‐date the published protocols have not improved upon the results of publications without their inclusion.^[^ ^34^, ^44^ ^]^

Preventing the immune rejection of these cells after transplantation is a current major hurdle to clinical translation. While autologous SC‐islets could be generated via induced pluripotent stem cells or using somatic cell nuclear transfer,^[^ ^45^, ^46^ ^]^ the cost and hassle of producing current good manufacturing practice (cGMP) compliant cells^[^ ^47^ ^]^ for each individual patient is a significant barrier to early use in clinical trials compared to an allogeneic hPSC line.^[^ ^48^ ^]^ Instead, encapsulation of insulin‐secreting cells, providing a selectively permeable physical barrier to convey immune protection, has been proposed as a solution.^[^ ^49^ ^]^ These approaches have been applied to SC‐islets but to date have not been designed to adapt to the peri‐implant environment temporally. Alginate hydrogel chemicals modified to reduce foreign body response (FBR) has been used successfully with SC‐islets to treat mice.^[^ ^50^ ^]^ Additionally, alginate hydrogel encapsulation of SC‐islets has been combined with the chemokine CXCL12 to also lessen the FBR in mice.^[^ ^51^ ^]^ Conformal coatings of SC‐islets, to lessen the diffusional distance of oxygen and nutrients by protecting cells with a thin layer of encapsulating material, has also successfully treated mice.^[^ ^52^ ^]^ In addition to immune protection, other recent work indicates that material properties may be important for the differentiation and maturation of SC‐islets.^[^ ^34^, ^53^ ^]^ An alternative to encapsulation to convey immune protection of transplanted SC‐islets is genetic modulation of the cells to render them hypoimmunogenic.^[^ ^54^ ^]^ This approach has recently been applied to SC‐islets by the genetically engineered overexpression or interferon‐*γ* induced expression of the cell surface protein programmed death‐ligand 1 (PD‐L1) in mice.^[^ ^32^ ^]^ These studies indicate that many avenues potentially exist for clinical transplantation of SC‐islets. Genetic engineering to introduce a drug‐inducible “kill‐switch” for transplanted SC‐islets and other cell types in the event that problems arise, such as the formation of a tumor,^[^ ^55^, ^56^ ^]^ is an additional approach being investigated.^[^ ^57^, ^58^ ^]^ Removable, selectively permeable macroencapsulation devices have also been developed for similar safety purposes to facilitate removal of the graft.^[^ ^59^ ^]^

## 3 Macroencapsulation Devices for SC‐Islets

**Figure 3:**
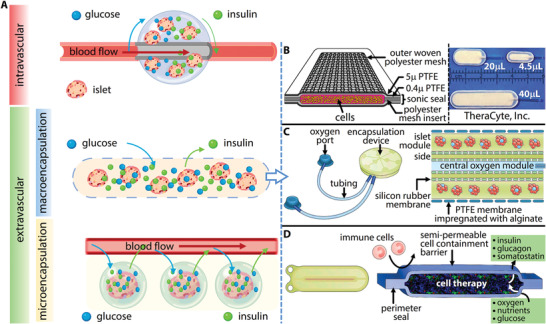
An overview of the hierarchy of islet encapsulation devices and some examples of commercially available macroencapsulation devices. A) Islet encapsulation methods can be categorized into intravascular or extravascular. Extravascular encapsulation techniques include macroencapsulation (the focus of this paper) and microencapsulation. B) The TheraCyte device. Adapted with permission.^[^ ^62^ ^]^ Copyright 2014, Frontiers in Bioscience. C) The Beta‐O_2_ device with ports for recharging oxygen and the encapsulation device. The central module can be filled with oxygen to supply the islet modules which are islets contained in a membrane. The membrane is made of PTFE impregnated with alginate to prevent infiltration of cells. Adapted with permission.^[^ ^49^ ^]^ Copyright 2017, Springer Nature. D) Schematic of the ViaCyte device, a refillable encapsulating system. The membrane prevents immune cells from entering but allows oxygen, nutrients and glucose to enter the reservoir and allows hormones to exit. Adapted with permission.^[^ ^49^ ^]^ Copyright 2017, Springer Nature.

Advances in the development of unlimited sources of insulin‐producing cells discussed in the previous section hold realistic promise for the future widening of SC‐islet transplant therapy. However, significant scientific hurdles still need to be overcome before SC‐islet replacement can become a viable treatment option for all people with T1D. As it is now recognized that traditional islet transplantation via infusion through the portal vein is a major contributor to transplanted cell death,^[^ ^10^ ^]^ research has focused on more favorable extra hepatic engraftment sites and encapsulation of islets. There are a number of methods for islet encapsulation (**Figure** **3**A) including intravascular methods where islets are anastomosed to the vessel, and extravascular techniques where implant is outside the bloodstream. Depending on the scale of the encapsulation materials, extravascular encapsulation can be categorized into: a) microencapsulation: techniques where only one or a few islets are coated with a permselective biopolymer, or b) macroencapsulation: systems where ≈10^3^–10^6^ islets are enclosed in a device that contains a semipermeable barrier. For the scope of this progress report, we focus on the latter. Each method provides immunoprotection of transplanted cells while allowing free diffusion of glucose and insulin. In macroencapsulation devices, immunoprotection is achieved by a selectively permeable membrane which impedes the movement of immune cells and immune factors (immunoglobulins) into the device,^[^ ^61^ ^]^ while allowing the free diffusion of oxygen, nutrients, insulin, and glucose to and from the encapsulated cells (Figure 3B–D).Immune cells can be readily blocked due to their large ≈10 µm diameter; however, large antibodies (IgM) and complement proteins (C1q) could only be hindered at pore diameters of ≈30 nm, although this may be less important if using an allogeneic stem cell source. This device must support the long‐term viability of the transplanted cells by providing a suitable environment (sufficient vascularization and oxygenation, minimal fibrosis, adequate cell spacing) for the safeguarding of efficacious islet cell function.

## 4 Sustaining SC‐Islet Viability in a Temporally Dynamic Environment

A primary design consideration of macroencapsulation devices is the ability to deliver a sufficient cell number to reverse T1D, while overcoming issues associated with cell overcrowding. The single most important device requirement is that the device supports cell viability. This is a significant challenge as the needs of encapsulated SC‐islets evolve progressively as they mature following transplantation (Figure 1). The transplantation process may involve transport of the cells to the clinic, preparation of the cell product and delivery into the device. Cell transport is not without its challenges as differentiated primary *β*‐cells do not proliferate, cannot be transported easily, and cryopreservation has not been evaluated.^[^ ^63^ ^]^ In the clinic, the insulin‐producing cells must be delivered to the implanted device without damage. Minimizing cell death during transplantation is imperative not only to ensure adequate insulin production, but also to minimize the production of biomolecules such as danger associated molecular patterns (DAMPs), which are released upon cell death and can activate immune cells,^[^ ^64^ ^]^ leading to a heightened FBR toward the implant. SC‐islets can be preloaded into the device prior to implantation or can be delivered minimally invasively following a device integration phase.^[^ ^49^, ^65^ ^]^ SC‐islets have high metabolic needs and require a rich vascular network to provide adequate oxygen supply for cell survival.^[^ ^66^ ^]^ The survival of SC‐islets is highly dependent upon oxygen and nutrient transfer in the stages immediately following transplantation. In fact, hypoxia is known to be a major underlying factor responsible for the loss of islet mass, viability, and function before, during and after islet transplantation.^[^ ^67^ ^]^ As SC‐islets developed in vitro have been shown to be transcriptionally and functionally immature exhibiting drastic changes and maturation when transplanted in vivo,^[^ ^16^ ^]^ the needs of the cells evolve as the cells mature. The peri‐implant tissue is also temporally dynamic (Figure 1), as discussed later in this section. The macroencapsulation device must support the viability of the transplanted cells at all stages throughout the maturation process of the cells at the transplantation site.

### 4.1 Transport across Macroencapsulation Device and Retrievability

Transport of insulin out of a macroencapsulation device is a critical design parameter, as insufficient diffusion of insulin into the blood in response to hyperglycemia is a failure of the device to adequately treat T1D. The diffusion of both glucose into the device and insulin out are time limited factors that must be accounted for in the device permeability. Additionally, once a device is implanted a complex series of events comprising the FBR is initiated, which can result in the formation of a fibrous capsule around the device, to protect the host from this foreign object. The fibrotic capsule, as discussed in Section 6, is very damaging to the success of devices for T1D as it can obstruct diffusion, compounding these challenges (Figure 1). Macroencapsulation devices provide the dual possibility of immunoprotecting transplanted SC‐islets and thereby removing the need for long‐term immunosuppression, while also being retrievable in the case of adverse events, which is vital for regulatory approval and successful clinical translation.^[^ ^68^ ^]^ Recent work has described retrievable implants for xenogeneic cells, which alleviates the need for long‐term immunosuppression.^[^ ^69^, ^70^ ^]^ This is especially important in current and future clinical trials with stem cell derived cells, where tumorigenicity is a crucial concern.^[^ ^55^ ^]^

### 4.2 Device Geometry Considerations

A critical parameter in the design of macroencapsulation devices is the device geometry. The device must be large enough to achieve a sufficient cell dose to reverse T1D, whilst overcoming issues associated with cell density and overcrowding. In order to evaluate the geometric considerations of a device to achieve a therapeutic dose, a thickness of 0.4–0.6 mm can be assigned to the device. This thickness range is appropriate as cells should not be greater than 0.2–0.3 mm, from the targeted permeability membrane (unless a method of oxygenation is incorporated) to allow sufficient transport of vital oxygen and nutrients, if the ingrowth of vasculature is inhibited.^[^ ^64^, ^70^, ^71^, ^72^, ^73^, ^74^ ^]^ It can also be assumed that 1 IEQ corresponds to the tissue volume of a perfectly spherical islet with a diameter of 150 µm.^[^ ^71^ ^]^ Islet density has been recommended to be 5–10% of the volume fraction of the device.^[^ ^72^ ^]^ Based on these inputs, the number of islets that can be delivered per mm^3^ in a macroencapsulation device (1 × 1 × 0.6 mm) is 11.32–33.97 IEQ mm^−3^ (11.32–33.97 IEQ µL^−1^). The clinical therapeutic dose as per the Edmonton protocol is 10 000 IEQ kg^−1^. The number of cells needed to achieve this dose is 700 000 IEQ for a person of 70 kg weight. In the case of 0.6 mm thickness and 10% volume fraction (33.97 IEQ µL^−1^) the volume required to achieve a therapeutic cell dose is 20.6 mL. If we do the same calculation for a 0.4 mm thickness and 5% volume fraction (11.32 IEQ µL^−1^), the volume required is 61.8 mL. This calculation underpins a major challenge in the delivery of a therapeutic dose of islets in encapsulation devices. It must be noted, however, it is now estimated that only 3000–5000 IEQ per kg remain viable following intra‐portal transplantation of 10000 IEQ per kg via the Edmonton protocol,^[^ ^12^ ^]^ therefore 3000–5000 IEQ per kg may be more representative of the dose required.

A number of macroencapsulation devices have reached clinical trials, including Beta‐O_2_ Technologies Ltd *β*‐air, and the ViaCyte PEC‐Encap (see Figure 3). These devices vary greatly in their appearance, with *β*‐air having a circular geometry and PEC‐Encap being rectangular. While information on exact dimensions and cell loading data is not publicly available, it is clear that a large number of cells (and resulting volume) is required to reach an adequate therapeutic dose to reverse T1D, with companies often taking the approach of implanting multiple devices per patient. 2–6 PEC‐Encap devices have been implanted per patient (NCT02239354), compared to 1–2 *β*‐air devices.^[^ ^1^ ^]^ The source of transplanted cells also varies between each device, with Beta‐O_2_ Technologies Ltd using allogenic human islets, whilst ViaCyte remains the only group using stem cell derived (embryonic stem cell line) pancreatic progenitor cells in clinical trials for T1D.^[^ ^3^, ^25^ ^]^ However, recent announcements from Vertex Pharmaceuticals Inc. indicate that they will begin a Phase I/II clinical trial in early 2021 for VX‐880, the first allogenic human stem cell‐derived islet cell therapy product.

### 4.3 Maintaining Viability throughout a Tiered Foreign Body Response

As the peri‐implant environment is evolving, it is challenging to promote and maintain the survival of transplanted islets for a prolonged time period.^[^ ^75^ ^]^ It is critical to minimize trauma at all stages, beginning with surgical implantation of the device and continuing to the delivery and subsequent maturation of cells. Implantation of a macroencapsulation device is a 3‐tier trauma including: the surgical procedure; the chemistry and size of the implanted device; and the type and number of transplanted cells.^[^ ^64^ ^]^ Inevitably, any surgical procedure will initiate a tissue repair process which is aggravated by inserting an artificial device and may be further intensified if the device contains cells. Furthermore, T1D is a disorder that is characterized by an autoimmune response, and the transplantation process and cell delivery can further induce immune responses that can compromise engraftment and function.^[^ ^49^ ^]^ Tissue damage caused during implantation of a medical device initiates a wound healing response that is initially characterized by spontaneous protein adsorption to the implant surface. The presence of fibrinogen and other proteins contributes to the formation of a dense fibrin network, which subsequently promotes adhesion of leukocytes and activates phagocytes to secrete cytokines and chemokines such as IL‐1, TNF*α*, VEGF, and IL‐8.^[^ ^76^ ^]^ This attracts leukocytes to the implant surface and activates angiogenesis in the vicinity of the implanted device. Activated macrophages bind to adsorbed proteins on the implant and fuse to form multinucleated giant cells and further release inflammatory cytokines, signaling myofibroblasts to synthesize procollagen. The maturation of procollagen and other extracellular matrix proteins contributes to the formation of a dense fibrous capsule, which is impermeable to many compounds.^[^ ^77^ ^]^ The formation of a fibrous capsule around a macroencapsulation device therefore presents a twofold problem: i) the impermeability of this layer prevents diffusion of glucose and insulin required for device functionality^[^ ^78^, ^79^ ^]^ and ii) the diffusion distance between encapsulated islets is increased, with necrosis likely if islets are >300 µm from neighboring blood vessels.

### 4.4 Implant Site Considerations

A number of potential implant sites have been discussed in the literature for macroencapsulation devices for T1D: such as subcutaneous,^[^ ^64^, ^80^, ^81^ ^]^ intramuscular,^[^ ^68^ ^]^ peritoneal,^[^ ^15^, ^82^ ^]^ and omentum.^[^ ^83^ ^]^ The ability for minimally invasive delivery makes the subcutaneous sites an attractive option for device implantation, but success has been limited due to the low vascular density of this space.^[^ ^64^, ^84^ ^]^ Intramuscular implant sites are promising in this regard as they a have higher affinity for neovascularization,^[^ ^85^ ^]^ and can be accessed minimally invasively with specialized delivery devices.^[^ ^86^ ^]^ The intraperitoneal space has also been explored as a potential site of implantation, as the peritoneal cavity can accommodate large volumes of cells that are required to reverse T1D in patients. However, accessing this space can result in numerous complications associated with intra‐abdominal procedures. Additionally, the implant site affects the biomechanics of the device as different external forces are exerted in different anatomical locations that ultimately may limit the functionality, performance, and lifetime of the device. Furthermore, the device can impose chronic mechanical loading and disrupt the surrounding tissue, which induces tissue remodeling and can elicit the FBR.^[^ ^78^ ^]^ Currently, there has not been consensus on the optimal anatomical location for these devices. As implant success is hindered by fibrosis of the implant, impairing nutrient transport and leading to cell death,^[^ ^84^ ^]^ choosing an implant site that minimizes the effects of this tissue repair process and the ultimate formation of a dense fibrous capsule surrounding the implant will be imperative in establishing an optimal environment for cell survival and device efficacy.

### 4.5 Clustering Maturation

Whilst the extracapsular environment is highly volatile during the early stages of device implantation and cell delivery, the behavior of cells encapsulated within the device is also temporally dynamic. Following transplantation, SC‐islets cells endure significant transcriptional changes before maturation to closely resemble adult *β*‐cells.^[^ ^87^ ^]^ Fetal SC‐*β*‐cells are polyhormonal, expressing glucagon and somatostatin in addition to insulin.^[^ ^3^ ^]^ Additionally, these cells respond to multiple secretory stimuli other than glucose.^[^ ^22^ ^]^ As a result, these cells exhibit weak glucose‐stimulated insulin secretion, and acquire glucose responsiveness only through functional maturation.^[^ ^39^, ^88^ ^]^ Endocrine cell clustering, as occurs in the native pancreas, is essential for islet maturation.^[^ ^33^ ^]^ Whilst significant progress has been made toward simulating this process in vitro,^[^ ^33^ ^]^ the extra‐ and intra‐capsular environment of SC‐*β* cell macroencapsulation devices remains to be optimized to encourage and support this maturation step in vivo, post‐implantation.

### 4.6 Encouraging Vascularization

Encouraging vascularization at the implant site is critical to promote cell survivability following cell delivery. Cell death and graft failure results from the low oxygen tension created by delayed and insufficient vascularization.^[^ ^89^ ^]^ This is particularly consequential for *β*‐cells, which are highly metabolic and will reduce insulin production under low oxygen tension.^[^ ^49^ ^]^ In addition to improving oxygenation at the implant site, vascularization is also required for rapid insulin release kinetics and removal of metabolic waste.^[^ ^79^ ^]^ Prevascularization of the implant site/encapsulation device can be used to prepare the site for delivery of cells, allowing a sufficient vascular network to be established prior to cell loading. This process also decouples the implant site trauma of device implantation and cell delivery. Extensive research has focused on optimizing the extra‐capsular environment to promote cell survival and long‐term device function (discussed in Section 5.1), with a limited understanding of the effect of the intra‐capsular environment on the encapsulated cells. The intra‐capsular environment likely plays an important role in cell survival, for example, immediately following encapsulation, islets undergo a cellular transition during which *β*‐cells are sensitive to changes in the rigidity of the microenvironment.^[^ ^64^ ^]^

### 4.7 Incorporating ECM Inside the Capsule

Incorporation of a suitable extracellular matrix (ECM) within a macroencapsulation device can promote survival of encapsulated cells. Interactions between cells and the ECM can mediate insulin release and cell proliferation and can protect cells from released cytokines, helping to conserve cell viability.^[^ ^14^ ^]^ In the native pancreas, cell‐cell communication between islets is essential to coordinate insulin release, and insulin production per cell has been shown to increase with three‐dimensional organization.^[^ ^49^, ^90^ ^]^ Islets which retain their native ECM following isolation have also exhibited reduced apoptosis rates and improved insulin response in vitro compared to purified islets.^[^ ^91^ ^]^ Collagen type IV is thought to be an essential ECM component to promote islet functionality, although the mechanisms underlying islet‐collagen IV interactions is poorly understood.^[^ ^91^ ^]^ Llacua et al. found that islets encapsulated within 3.4% purified alginate with 50 µg mL^−1^ collagen type IV at a ratio of 800 islets per mL enhanced their glucose induced insulin secretion (GIIS) at least two‐fold in comparison to islets encapsulated in alginate only. The same study observed that the addition of laminin sequences 0.01 mM Arginine‐Glycine‐Aspartate (RGD), 1 mM Leucine‐Arginine‐Glutamate (LRE), and 0.01 mM Pro‐Asp‐Ser‐Gly‐Arg (PDSGR) in combination with 50 µg mL^−1^ collagen type IV better maintained GIIS than islets encapsulated without ECM components in vitro, with the combination of 1 mM LRE with 50 µg mL^−1^ collagen type IV having the most pronounced effect.^[^ ^92^ ^]^ The same authors found that encapsulation of islets with these ECM components reduced cell necrosis following exposure to TNF‐*α*, IFN‐*γ*, and IL‐1*β* cytokines, compared to a control group with no ECM.^[^ ^93^ ^]^ Alginate capsules have also been used to generate a cell‐sustaining environment for encapsulated cells. More rigid alginate capsules (2%, high‐G alginate) were found to impair cell growth compared to less rigid capsules (3.4%, intermediate‐G alginate).^[^ ^94^ ^]^ Optimizing the design of the intra‐capsular environment to closely mimic the native ECM will be an important step toward protecting transplanted cells through the vulnerable stages of their maturation and will ultimately enhance overall device functionality.

## 5 Circumventing Islet Hypoxia

While vascularization at the implant site promotes survivability of cells, hypoxia and necrosis at the center of islet clusters following transplantation is a concern, as intra‐islet vessel development is limited by cell encapsulation.^[^ ^79^ ^]^ Large volumes of encapsulated islets are required to accommodate sufficient insulin production;^[^ ^4^, ^65^, ^95^ ^]^ however, large delivery volumes can aggravate mass transport complications resulting in central necrosis of islet clusters. Successful clinical translation of macroencapsulation devices for SC‐islets will require increasing islet density to reach clinically adequate insulin production, and islet densities can be based on the work with cadaveric islets. Previously, it has been shown that islet loading at a surface density of 2250 IEQ cm^−2^ with no external oxygen supply sustained normoglycemia for a period of only 1–2 days post implantation. In contrast, a device loaded at a surface density of 2300 IEQ cm^−2^ was shown to maintain normoglycemia for a period of 58 days when an exogenous supply of oxygen was delivered to the cells daily.^[^ ^96^ ^]^ Evron et al. describe loading up to 4800 IEQ cm^−2^ in an ultrapure high‐guluronic acid alginate mixture into their *β*‐air device prior to implantation in diabetic rats, with an average recovery of initial oxygen consumption rate of 88% indicating minimal loss of viable tissue during implantation.^[^ ^70^ ^]^ This highlights that oxygen is a critical limiting factor in transplanted islet survival and that future efforts to implant encapsulated islets should aim to address islet oxygenation in vivo. This is particularly pertinent for translation of macroencapsulation devices from small rodent studies to large animal and clinical studies, where a larger volume of encapsulated islets is required, reducing the surface area to volume ratio and exacerbating mass transport complications.

Hypoxia plays a significant role in islet viability and functionality prior to, during, and after islet transplantation.^[^ ^97^ ^]^ *β*‐cells are particularly sensitive to hypoxia and they need to be exposed to a highly oxygenated environment to survive.^[^ ^98^ ^]^ It has been demonstrated that even if *β*‐cells survive the hypoxic environment during transplantation, their function may be impaired (when pO_2_ < 40 mmHg)—an effect that can persist even after re‐oxygenation/revascularization.^[^ ^99^, ^100^, ^101^, ^102^, ^103^, ^104^ ^]^ Therefore, post‐transplantation graft survival is completely reliant on diffusion of oxygen (O_2_) and nutrients from the surrounding environment of the implant. Several in vivo and in silico diffusion‐reaction models have demonstrated how the oxygen environment and islet size are the primary limiting factors for islet survival,^[^ ^105^, ^106^, ^107^ ^]^ with average‐sized human islets (150 µm diameter) presenting an hypoxic core under normal culturing conditions (21% O_2_). Increasing oxygen tension up to 350 mmHg (47% O_2_) reduced the hypoxic core formation and increased cell viability, even for larger islet cells.^[^ ^105^ ^]^ As seen in islets, these hypoxia‐related issues will likely remain in SC‐*β* cells. Faleo et al. demonstrated that more than half of SC‐islets die shortly after transplantation in mice, due to nutrient and oxygen deprivation (ischemia).^[^ ^108^ ^]^

**Figure 4:**
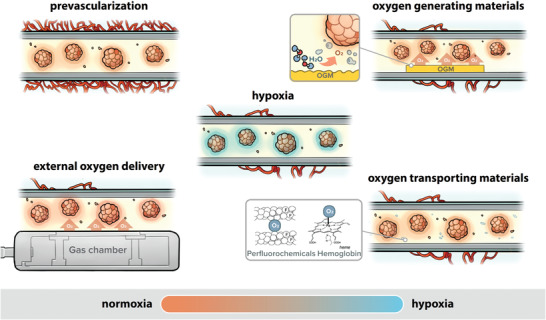
Schematics showing the different methods of oxygenation to mitigate hypoxia in macroencapsulation devices: prevascularization, external oxygen delivery, incorporation of oxygen generating material (OGM), or oxygen transporting materials.

The supply of oxygen to macroencapsulation devices relies on several interdependent factors including encapsulant or membrane permeability toward oxygen, the spatial arrangement of host vasculature at the implant site, rate of oxygen consumption of the encapsulated cells, tissue density, local pO_2_ levels at the implantation site, and geometry of implant.^[^ ^109^ ^]^ Consequently, to overcome these limitations, enhanced graft oxygenation is key to maintaining cell viability and functionality after transplantation. Two major strategies can be considered: i) increase of the vasculature surrounding the device prior to cell encapsulation, enhancing the blood supply to the graft and ii) oxygen delivery to the encapsulated cells through oxygen generating materials, oxygen transporting materials or external oxygen generators (**Figure** **4**).

### 5.1 Prevascularization

Following implantation of a medical device, neovascularization occurs slowly, at a rate of ≈5 µm h^−1^,^[^ ^110^ ^]^ and it can take many weeks/months for an implant to become surrounded by a dense vascular network.^[^ ^111^ ^]^ The survival of *β*‐cells is highly dependent upon oxygen and nutrient transfer in the stages immediately following transplantation, and if this is not achieved the cells will likely die by hypoxia.^[^ ^75^ ^]^ Prevascularization of the implant site has been explored to prime the site for cell implantation, thereby promoting cell survival immediately following transplantation. A number of approaches to prevascularization have been examined which include implantation of an empty macroencapsulation device followed by cell delivery after a number of weeks or months,^[^ ^66^, ^112^ ^]^ and implantation of a removable construct to prime a subcutaneous device‐less space for islet transplantation.^[^ ^113^ ^]^ Preimplantation of a device for a period of 3 months prior to islet implantation in a rodent study has been shown to increase diabetic cure rates, with 6/6 animals cured using pre‐implanted devices, compared to 1/6 in freshly implanted devices.^[^ ^112^ ^]^ A six week pre‐vascularization period has also been shown to provide sufficient oxygenation to an implanted device in a rat model, where oxygen tension was measured with microelectrodes polarized at −0.8V. pO_2_ measurements of pre‐vascularized devices were 4.4–12.2% (equivalent to 32.66–90.55 mm Hg, *n* = 14), with most readings exceeding 5% (37.1 mm Hg), which is comparable to the mean pO_2_ measured within vascularized islets in the native pancreas.^[^ ^15^, ^114^ ^]^

Rapid vascularization in the vicinity of devices can be promoted by the inclusion of growth factors, such as vascular endothelial growth factor (VEGF) and basic fibroblast growth factor (bFGF), to facilitate the controlled release of angiogenic cues and stimulate angiogenesis.^[^ ^115^, ^116^ ^]^ Uncontrolled expression of VEGF, however, can lead to abnormal vascular growth and vascular tumors, while short‐term expression leads to unstable vessels, which promptly regress following the removal of the angiogenic stimulus. The formation of a functional vascular environment is dependent upon both the generation of new vessels and the stabilization of these vessels, which requires the recruitment of smooth muscle cells and ECM deposition.^[^ ^115^ ^]^ Platelet‐derived growth factor (PDGF) is a key mediator of vessel maturation through recruitment of smooth muscle cells,^[^ ^76^ ^]^ in addition to TGF‐*β* which stimulates ECM production.^[^ ^117^ ^]^ Therefore, a combination of pro‐angiogenic and pro‐maturation growth factors may improve the quality of vascular networks in the vicinity of medical implants. Kastellorizios et al. showed that a combination of VEGF and PDGF at a 2:1 ratio in dexamethasone‐releasing poly(lactic‐co‐glycolic acid) (PLGA) microspheres increased angiogenesis and improved capillary orientation compared to PLGA microspheres with dexamethasone only or PLGA microspheres with both dexamethasone and VEGF in subcutaneous tissue in a rat model.^[^ ^118^ ^]^ Furthermore, the same authors have observed that inclusion of dexamethasone in PLGA microspheres have been shown to prevent the acute phase of the foreign body reaction to subcutaneous implants in a large animal model.^[^ ^119^ ^]^ In addition to VEGF and PDGF, bFGF is also known to stimulate angiogenesis. Luan and Iwata investigated transient implantation of agarose rods with bFGF and heparin to generate prevascularized pockets for subcutaneous islet transplantation in a diabetic rat model.^[^ ^120^ ^]^ Following implantation of agarose rods (4 mm diameter, 2.5 cm length) with bFGF‐heparin (50 and 25 µg per rod, respectively) for 1 week, a thick vascularized pocket formed from which the agarose rods were removed, and 1500 islets were allogeneically transplanted to the prevascularized subcutaneous pockets. 9 of 10 rats exhibited stable normoglycemia for >100 days, compared to non‐normalization of blood‐glucose in rats that received islets subcutaneously but without prevascularization.

Vascularization can also be promoted by microarchitecture of the implant surface, not requiring the use of exogenous agents. Vascularizing membranes, bearing 5 µm pores that allows host cells penetration and induce vascularization, have been incorporated in macroencapsulation devices such as TheraCyte (Baxter Healthcare, Figure 3B).^[^ ^80^, ^121^ ^]^ TheraCyte maintained cell survival and reversed diabetes in mice models after allogeneic^[^ ^5^, ^112^ ^]^ or xenogeneic^[^ ^122^ ^]^ islet transplantation. This device has been recently used in combination with bone marrow derived insulin producing stem cells, achieving an improved glycemic control in a diabetic rat model.  Another relevant example is Sernova Cell Pouch, which employs non‐biodegradable polymers to form a scaffold that allows infiltration with tissues and microvessels. Islets transplanted in this device reversed diabetes long term (>100 days) in a diabetic mice model, compared to subcutaneously implanted naked islets.^[^ ^123^ ^]^ Khosravi et al. investigated the effect of surface topography on neovascularization in a rodent model, and found that more complex topographical surfaces promoted an increased rate of neovascularization and vascular maturation compared to a relatively smooth surface.^[^ ^124^ ^]^ Although this work focused on titanium based materials most commonly used in orthopedic applications, surface roughness has also been shown to be advantageous for soft‐tissue applications, with surface roughness influencing protein adsorption, cell differentiation, and the FBR to implants.^[^ ^79^ ^]^ Skrzypek et al. describe a method of prevascularizing an islet macroencapsulation device in vitro using micropatterned poly‐ ethersulfone/polyvinylpyrrolidone (PES/PVP) membranes in a co‐culture of human umbilical vein endothelial cells (HUVECs) and human dermal fibroblasts (NHDFs). Micropatterned membranes showed clear cell orientation and cell interconnectivity, in comparison to non‐patterned membranes which showed no specific cell orientation.^[^ ^66^ ^]^ The co‐culture also formed stable endothelial networks, verifying that it closely replicates the in vivo environment. In vitro prevascularization has the potential advantage of reducing the number of surgeries required per patient, as the in vivo prevascularization period prior to cell delivery is greatly reduced. However, this technique is in preliminary stages and will require comprehensive optimization before successful clinical translation. The promotion of blood vessel formation for therapeutic purposes remains a challenge as physiological angiogenesis is a complex and highly concerted process.^[^ ^116^ ^]^

### 5.2 Oxygen Delivery

Prevascularization strategies are an interesting approach to provide an extra oxygen supply to the encapsulated islets/*β*‐cells. However, the immunoprotective membrane of macroencapsulation devices prevents the ingrowth of vessel, limiting the nutrient supply to only diffusion through the semipermeable membrane. The resulting hypoxic environment restricts the density of viable cells that can be loaded within the device, resulting in large or multiple devices required to achieve a therapeutic dose of cells.^[^ ^125^ ^]^ To mitigate this problem, inclusion of an oxygen delivery technology within the encapsulation device has been deeply investigated. Three main approaches have been considered: i) oxygen generating materials, ii) oxygen transporting materials, and iii) external oxygen delivery (Figure 4).

#### 5.2.1 Oxygen Generating Materials

Oxygen generating materials (OGMs), typically peroxides, can produce and deliver O_2_ by means of a chemical reaction that involves the formation and subsequent decomposition of hydrogen peroxide (H_2_O_2_). However, peroxide hydrolysis produces toxic by‐products (H_2_O_2_) and reactive oxygen species (ROS) that can compromise cell viability. Inflammatory damage can be overcome by the use of catalase as antioxidant agent.^[^ ^126^ ^]^ OGMs aim to provide a gradual and prolonged O_2_ release to cells, which can be controlled by integrating them into constructs such as scaffolds,^[^ ^127^ ^]^ nanofibers,^[^ ^128^ ^]^ hydrogels,^[^ ^129^ ^]^ or microspheres.^[^ ^127^ ^]^ Oxysite is a promising technology consisting of a self‐sustaining CaO_2_‐PDMS disk that can provide a controlled O_2_ supply to encapsulated cells.^[^ ^130^ ^]^ The Oxysite disk was incorporated into an immunoisolating agarose macroencapsulation device that can support elevated cell loading densities (1500 IEQ, or 8.5 × 10^6^ MIN6 cells cm^−3^). Islets and *β*‐cells encapsulated in this oxygen‐enhanced device showed increased survival and functionality (as seen by hyperglycemia correction), under hypoxic conditions for 30 days in a rodent diabetes model, while maintaining high cyto‐ and bio‐compatibility.^[^ ^131^ ^]^ To enable clinical translation, efforts to prolong the oxygen durability of Oxysite are ongoing, with a view to creating a scaled‐up version for clinical studies that, coupled with an unlimited cell source, could achieve longer implantation periods (>30 days).

The limited longevity of OGMs, as well as the potential biotoxicity of their degradation products are the main challenges for these technologies. OGMs that can provide oxygen for extended periods (months/years) would require prohibitively large devices. An interesting approach could be the use of refined systems to control oxygen release kinetics in response to the environmental oxygen levels.^[^ ^132^ ^]^ These “smart” oxygen‐responsive systems, based on H_2_O_2_/PVP/catalase microspheres bearing a 2‐nitroimidazole oxygen‐responsive shell, demonstrated an improvement in the survival of mesenchymal SC in vitro and in vivo.^[^ ^132^ ^]^

#### 5.2.2 Oxygen Transporting Materials

Materials that can solubilize and transport higher volumes of oxygen while enhancing oxygen permeability within capsules have been investigated. These materials—artificial oxygen carriers (AOCs)—were initially developed as artificial blood substitutes but their application has shifted toward “oxygen therapeutics”, including oxygen delivery to encapsulated cells.^[^ ^133^ ^]^ Two main families of AOCs can be distinguished: i) perfluorocarbon (PFC) based oxygen carriers, and ii) hemoglobin‐based oxygen carriers (HbOs).

PFCs are a family of compounds that exhibit very high oxygen solubility (20‐fold volume percent oxygen solubility compared to water) and favorable diffusion characteristics.^[^ ^134^, ^135^ ^]^ Owing to their unique characteristics, PFCs have been used in different applications, including organ preservation,^[^ ^136^, ^137^ ^]^ artificial blood substitutes,^[^ ^138^ ^]^ or oxygen delivery to cells. PFC‐based oxygenated scaffolds (perfluorodecalin (PFD) emulsions,^[^ ^139^ ^]^ PFD‐alginate capsules^[^ ^140^ ^]^) reduced hypoxia and cell death (reducing ROS generation) in encapsulated stem cells,^[^ ^141^ ^]^ *β*‐cells,^[^ ^139^ ^]^ and pancreatic islets, both in culture^[^ ^139^, ^142^, ^143^ ^]^ and implanted in mice.^[^ ^140^ ^]^ PFCs can be pre‐loaded with oxygen prior cell encapsulation, proving an additional initial oxygen supply. Additionally, once the initial oxygen payload has been delivered, the remaining PFCs could effectively increase the oxygen permeability inside the macroencapsulation device, improving cell nutrition. This enhancement in oxygen permeability has led to improved survival of encapsulated islets in alginate‐PFC systems, under hypoxic conditions.^[^ ^144^, ^145^ ^]^ Despite their promising properties, the high hydrophobicity of PFCs limits their incorporation within physiological fluids and their shelf stability. Moreover, some PFC‐based products have shown safety concerns associated with organ retention, and unwanted side effects usually related to phagocytosis uptake.^[^ ^135^ ^]^ Research must focus on producing emulsion‐based PFC technologies bearing low particle sizes (facilitate oxygen transport and evade phagocytosis), long term shelf stability (months/years) and having large‐scale manufacturing feasibility.^[^ ^135^ ^]^

Hemoglobin is the component of blood that transports oxygen by covalent bonding between oxygen and the heme group. The use of hemoglobin as an oxygen carrier is compromised by its low availability and portability, special storage requirements of blood products and risk of toxicity due to its low physicochemical stability.^[^ ^146^ ^]^ Consequently, research has been focused on developing synthetic or semisynthetic HbOs able to provide efficient oxygenation while improving their long‐term stability and biocompatibility. These systems have been described in detail elsewhere.^[^ ^147^ ^]^ A recent HbO based on marine hemoglobin, called HEMOXcell,^[^ ^148^ ^]^ demonstrated enhanced viability and multipotency of cultured human mesenchymal SC. The use of HEMOXcell to reduce cellular hypoxia was further investigated in an in vitro model of islet encapsulation, in comparison with a PFC derivative. Although both AOCs increased cell viability and decreased markers of hypoxia when cultured with rat islets at 2% pO_2_, HEMOXcell had the capacity to restore insulin secretion to normal levels after 24 h of incubation,^[^ ^149^ ^]^ due to its superoxide dismutase activity.^[^ ^150^ ^]^

Although AOCs have demonstrated great promise to provide an extra oxygen supply post transplantation, further evaluation in more clinically relevant encapsulation devices is required. AOCs are inherently limited by their long‐term stability (e.g., colloidal stability of PFD emulsions, tetramer disintegration in HbOs, etc.), unwanted side effects (potential inflammatory reactions), and storage challenges. Future developments in the design of AOC formulations, focusing on process standardization and scale‐up production are immediate priorities to further refine and validate the use of AOCs in regenerative medicine.

#### 5.2.3 External Oxygen Delivery

As an alternative to in situ oxygen generation/transporting, exogenous oxygen gas injection into a chamber within an immunoisolating device is a very promising approach. Several external oxygenation technologies have been developed in the last number of years.^[^ ^64^, ^151^, ^152^, ^153^ ^]^ In the quest to create a bioartificial pancreas, the *β*‐air device (Figure 3C) has emerged at the forefront. This device, developed by Beta‐O_2_, comprises of: i) an alginate hydrogel containing immobilized islets, ii) an immunoisolating semi‐permeable membrane comprised of two hydrophilized PTFE membranes with a 0.45 µm pores, iii) a rubber silicone membrane that separates the encapsulated islets from the iv) oxygen reservoir. This oxygen chamber is connected to subcutaneous access port for daily refuel with an O_2_ gas blend that could last for up to 30 h.^[^ ^64^ ^]^ Prior to clinical assessment, the *β*‐air device has shown efficacy in xenogeneic and allogeneic islet transplantation for the treatment of diabetes using rats,^[^ ^70^, ^152^ ^]^ pigs,^[^ ^154^, ^155^ ^]^ and non‐human primates.^[^ ^156^ ^]^ The *β*‐air device has been optimized to support high encapsulation surface densities (4800 IEQ cm^−2^) with an initial gas chamber pO_2_ of 570 mmHg (76% O_2_), showing nearly 90% preservation of islet viability post‐explanation.^[^ ^70^ ^]^ In a first‐in‐human trial performed in 2012, the *β*‐air device was able to maintain human donor islet survival and overcome immune rejection for 10 months in a patient with T1D. Due to the low islet dose used in this study (2100 IEQ kg^−1^) diabetes was not reversed and only modest improvements in the glycemic control were achieved. In a further clinical trial performed in 2019, the *β*‐air device was implanted in 4 diabetic patients for 3–6 months at 1800–4600 IEQ per kg body weight.^[^ ^13^ ^]^ Although the device promoted cell viability through O_2_ delivery, the functionality of transplanted islets was minimal. In order to improve patient compliance, a next‐generation device that aims to be refilled weekly is expected to enter clinical trials soon. These second‐generation devices will be adapted for stem cell clusters.^[^ ^157^, ^158^ ^]^

Finally, in order to avoid the oxygen refillability issues of the abovementioned technologies, and improve their long‐term functionality, a novel approach consisting of wearable electrochemical oxygen generators (wEOGs) has been proposed.^[^ ^125^, ^159^ ^]^ These wEOGs could continuously produce oxygen from water electrolysis and supply it to macroencapsulation devices, requiring smaller gas chambers that could reduce the size of these devices.^[^ ^125^ ^]^ This water‐refillable and battery‐powered technology could be coupled with oxygen enabled implantable cell encapsulation devices as is being investigated by the startup Procyon Technologies LLC in collaboration with Novo Nordisk to incorporate SC‐islets within this technology.^[^ ^160^ ^]^ A similar technology, developed by Giner Life Sciences integrates implantable electrochemical oxygen generators (iEOG) to produce and supply O_2_ to implantable immune‐isolating devices.^[^ ^161^, ^162^ ^]^ Proof‐of‐concept studies will define the feasibility of this technology.

## 6 Minimizing Foreign Body Response

### 6.1 Geometry, Topography, and Mechanical Actuation

**Figure 5:**
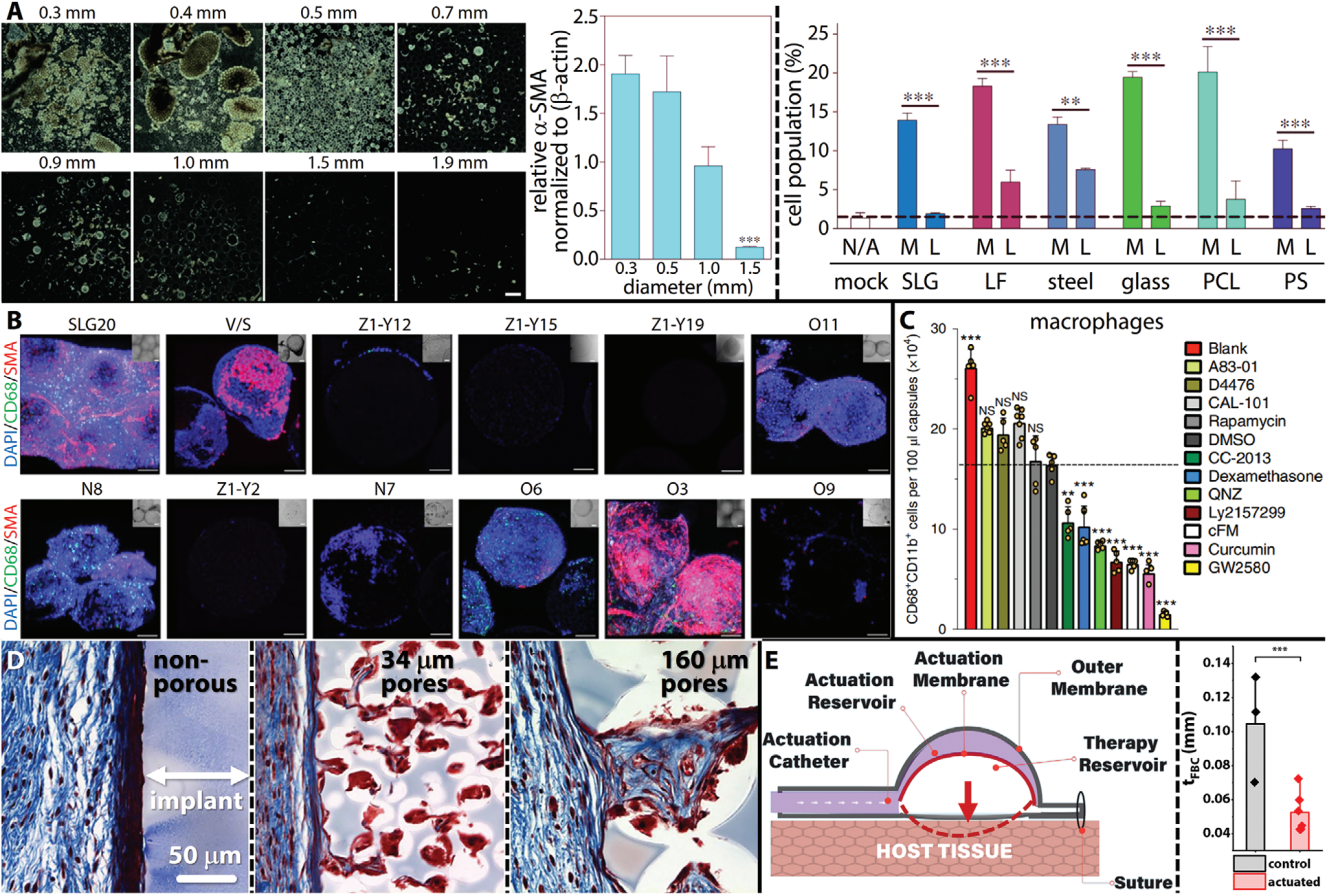
Strategies for minimizing fibrosis. A) Increase in the spherical diameter of materials, including hydrogels, ceramics, metals, and plastics, leads to reduced FBR. Scale bar is 2 mm. Adapted with permission.^[^ ^166^ ^]^ Copyright 2015, Springer Nature. B) Confocal microscopy images showing reduced fibrosis in modified alginate microcapsules, identified through a combinatorically developed hydrogel library. Scale bars are 100 µm. Adapted with permission.^[^ ^178^ ^]^ Copyright 2016, Springer Nature. C) Crystallized drug formulations for long‐term controlled‐release suppress FBR in both rodents and non‐human primates for at least 1.3 years and 6 months, respectively. Adapted with permission.^[^ ^179^ ^]^ Copyright 2019, Springer Nature. D) Porosity‐dependent FBR in poly(2‐hydroxyethyl methacrylate) implants. Collagen is shown in blue, cellular cytoplasm in red, and cell nuclei in black. Adapted with permission.^[^ ^171^ ^]^ Copyright 2013, Springer Nature. E) Soft robotic actuation applied to implanted devices reduces the average thickness across fibrotic capsule. Adapted with permission.^[^ ^176^ ^]^ Copyright 2019, American Association for the Advancement of Science.

Recent advances may provide mechanisms to reduce the foreign body response^[^ ^17^ ^]^ where mechanical and pharmacological strategies have been employed (**Figure** **5**). It is evident that the size and shape of the implant play a role in modulating the immune response.^[^ ^163^ ^]^ For example, less tissue response was found from implanting circular rods compared to triangular shapes,^[^ ^164^ ^]^ and smooth contoured implants showed reduced macrophage response compared to those with angular geometry.^[^ ^165^ ^]^ In terms of size, spheres greater than 1.5 mm in diameter showed mitigated FBR in rodents and non‐human primates (Figure 5A).^[^ ^166^ ^]^ Microgeometry and thickness also have important effects on FBR, with porous, thinner implants eliciting less of a fibrous capsule (Figure 5B,C).^[^ ^167^ ^]^ For other applications, groups have reported a correlation between porosity and macrophage switching to M2 anti‐inflammatory type; in a study related to cardiac tissue engineering pHEMA‐co‐MAA scaffolds with a 30 µm diameter pore size polarized macrophages to an M2 phenotype leading to a thinner fibrous capsule and enhanced neovascularization at 28 days post‐implant.^[^ ^168^ ^]^ Increasing the M2/M1 ratio is important to tissue remodeling and capsule thickness,^[^ ^169^ ^]^ and has been correlated with porosity in a number of other studies (Figure 5D).^[^ ^170^, ^171^ ^]^ Nanostructures on the surface can also modulate protein adsorption, and subsequently the immune response.^[^ ^172^, ^173^, ^174^ ^]^ Surface‐bound hydrophilic gold nanoparticles on the surface were shown to reduce immune‐complement activation by suppressing the activity of IgG.^[^ ^175^ ^]^ Our group has recently developed an innovative strategy to reduce fibrosis around an implanted device using the principles of soft robotics (Figure 5E). This approach uses mechanical actuation to actively modulate the biomechanics of the biotic‐abiotic interface by altering strain, fluid flow, and cellular activity in the peri‐implant tissue.^[^ ^176^ ^]^ We found that this actuatable device significantly improved the number of blood vessels (*p* = 0.0099; ≈400 CD31+ blood vessels mm^−2^, similar to native human islets^[^ ^177^ ^]^), reduced the thickness of the fibrous capsule formed (*p* = 0.0005, ≈two‐fold reduction) and improved the diffusion of a drug analogue through the formed fibrous capsule compared with non‐actuated controls in a subcutaneous rat model. There was no significant difference in macrophages present between groups (*p* = 0.6963) and a significant reduction in myofibroblasts (*p* = 0.0036) in the actuated group. We propose that actuation reduced the differentiation and proliferation of myofibroblasts and therefore extracellular matrix production.

### 6.2 Immunocloaking

Immunocloaking using extracellular matrix (ECM) is an alternative strategy that has been used to attenuate FBR,^[^ ^180^, ^181^, ^182^ ^]^ for example, polypropylene mesh covered by ECM showed a reduction in FBR, M1 macrophages and giant cells, with an increased M2/M1 macrophage ratio.^[^ ^183^ ^]^ Pharmacological solutions have been developed pre‐clinically to cloak the implant so that it becomes invisible to the body and does not initiate the FBR. One such approach targets macrophages (Figure 5C), a key player in initiation of the FBR, by using the small molecule GW2580 to inhibit cross‐phosphorylation and activation of the macrophage specific factor cytokine receptor colony stimulating factor‐1 receptor (CSF1R).^[^ ^179^, ^184^ ^]^ This GW2580 cloaking approach results in a reducing in the FBR to implants, but it is unclear how it affects vascularisation, as endothelial/macrophage crosstalk is critical for arteriogenesis.^[^ ^185^ ^]^ Additionally, it requires long‐term sustained release of GW2580, and the off‐target effects and long‐term complications have not yet been investigated. Kharbikar et al. have recently published an extensive review of various strategies and techniques that have been used for immunomodulation and prevention of fibrosis for macroencapsulation devices.^[^ ^78^ ^]^

### 6.3 Refillability

The ability to replenish SC‐islets in a macroencapsulation system is advantageous given the temporally dynamic environment of biohybrid artificial pancreas. Substantial cell loss can occur early after transplantation and late after islet infusion. Hence, multiple islet infusions are often required to reverse insulin dependence.^[^ ^186^ ^]^ Many of the commercially available^[^ ^5^, ^80^ ^]^ and research stage devices^[^ ^86^, ^176^, ^187^ ^]^ have incorporated features (tubing and self‐sealing subcutaneous or transcutaneous ports) to enable the repeated delivery of cell cargo. This design consideration is particularly relevant to allow dosing and cargo adjustment if one thinks about future incorporation of continuous sensing of implant function.

## 7 Ability to Monitor Implant Function over Time and Closed Loop Control to Adjust Implant/Cargo Parameters over Time

Continuous measurement of circulating biomarkers is enabled by an emerging class of biosensors.^[^ ^188^, ^189^ ^]^ Wearable continuous glucose monitors (CGM) have been clinically adopted in the T1D community, thus reducing the burden of patient self‐monitoring and ensuring that patients remain in a euglycemic range.^[^ ^190^, ^191^ ^]^ A state‐of‐the‐art CGM worn after graft delivery could help correlate graft function to physiologic cell released insulin responses and help tailor and taper exogenous insulin delivery as the SC‐islet graft matures over time.

Incorporation of implantable CGM sensors within macroencapsulation systems could enable continuous measurement of glucose inside the cell compartment of the encapsulation device as a surrogate measure of device integration, function, and corresponding host response (vascularization and fibrosis) over time. For example, dual measurements achieved with a patient worn and cell compartment CGM could help decipher glucose diffusion into the device post‐implantation and help ascertain the best timing for cell loading if a staged approach is required. Recent advances in fully implantable CGMs with data transfer direct to smart devices could make this a reality. Implantable CGMs (iCGMs) have been tested in large animals^[^ ^192^ ^]^ and humans,^[^ ^193^ ^]^ and are in current development for clinical approval.^[^ ^194^ ^]^ The current iCGM is designed for subcutaneous implantation and would require additional design changes to allow signal to be transported from within a macroencapsulation device to a data receiving smart device if the implant site was deep within the body.^[^ ^192^ ^]^ This may also change the energy requirements and the development of wireless energy transfer may be required to maintain long‐term function.

With the refillable and retrievable design considerations discussed earlier, the access port is advantageous in allowing assessment of cell compartment oxygen tension and pH before cell loading. This process could be included as an additional safety check to avoid any conditions that would exacerbate the subsequent immune response to the cell cargo, if danger signals were released from damaged cells in the peri‐implant tissue. The port could also allow cell biopsy during clinical development to allay any regulatory fears of tumor development. Recent advances in the development of cell‐based biosensors that give insight into cell and tissue function in vivo could be an attractive technology to measure SC‐islet implant function. Recently, an insulin producing cell line expressing a fluorescent calcium sensor was developed to study beta‐cell function in vivo. The bioresponsive cells, stably expressed the genetically encoded chameleon‐based fluorescent sensor YC3.6cyto and the work demonstrated that cells could respond to glucose when implanted in mice. If further developed, the cells themselves could become the biosensing element to longitudinally monitor the function of implanted cells using non‐invasive imaging or temporal sampling through the port.^[^ ^195^ ^]^ With this real‐time sensor‐based information, and a refillable device, one could envision a closed‐loop feedback system where the number of cells, and thus the amount of insulin secreted could be adjusted based on the dynamic peri‐implant environment, cell maturation and patient need. With this type of system, we could envision a move toward an integrated platform for restoring endocrine pancreas function and sensing relevant real‐time outputs of a macroencapsulation device or a biohybrid artificial pancreas.^[^ ^196^ ^]^

## 8 Conclusion and Perspective

In order to design an optimal macroencapsulation device for SC‐islets, functional requirements and design considerations will be built on the knowledge foundation from cadaveric islet macroencapsulation devices and adopt an interdisciplinary, design‐centric approach. Functional requirements for these devices will vary over time after implantation, and this variability should be factored into the design, or the design should allow for adjustability – likely in terms of refillability and retrievability. Both the cargo and the peri‐implant environment are temporally dynamic, and necessitate adaptive design features. Integration of continuous sensing into a macroencapsulation system will not only enable monitoring of glycemic levels, and patient‐specific response to cell delivery allowing personalized dosing, but could also serve to monitor implant/host interactions such as vascularization and fibrosis. Access ports allow monitoring of graft phenotype by biopsy if required, particularly during the clinical trial phases. This information could be fed back into the inputs for the system, allowing responsive adjustment of number of SC‐islets (in a replenishable device) or allowing on‐demand delivery of agents such as oxygen, anti‐fibrotic or pro‐vascularization pharmacologics, or biomechanical actuation to mitigate fibrosis. The ability to access the intra‐capsular environment, potentially via a microcatheter in the replenishment line, may also allow temporal sampling to establish post‐transplant maturation and allay any regulatory fears of tumorigenicity. With closed loop feedback, and potentially smart control over agent replenishment, one could envision an autonomously regulating biohybrid artificial pancreas that would significantly reduce T1D patient pain points such as cumbersome self‐monitoring of glycemic levels and long‐term immunosuppressants.

## Conflict of Interest

J.R.M. is an inventor on patents and patent applications relating to the differentiation of SC‐islets, a consultant for Sana Biotechnology, and the Chief Scientific Officer and co‐founder of Salentra Biosciences. G.P.D. is an inventor on patents and patent applications relating to macroencapsulation devices.

## References

[1] P.‐O. Carlsson, D. Espes, A. Sedigh, A. Rotem, B. Zimerman, H. Grinberg, T. Goldman, U. Barkai, Y. Avni, G. T. Westermark, L. Carlbom, H. Ahlström, O. Eriksson, J. Olerud, O. Korsgren, Am. J. Transplant. 2018, 18, 1735. 29288549 10.1111/ajt.14642 PMC6055594

[2] L. Velazco‐Cruz, J. Song, K. G. Maxwell, M. M. Goedegebuure, P. Augsornworawat, N. J. Hogrebe, J. R. Millman, Stem Cell Rep. 2019, 12, 351. 10.1016/j.stemcr.2018.12.012 PMC6372986 30661993

[3] A. Rezania, J. E. Bruin, P. Arora, A. Rubin, I. Batushansky, A. Asadi, S. O'Dwyer, N. Quiskamp, M. Mojibian, T. Albrecht, Y. H. C. Yang, J. D. Johnson, T. J. Kieffer, Nat. Biotechnol. 2014, 32, 1121. 25211370 10.1038/nbt.3033

[4] S. Song, S. Roy, Biotechnol. Bioeng. 2016, 113, 1381. 26615050 10.1002/bit.25895 PMC5873326

[5] M. Kumagai‐Braesch, S. Jacobson, H. Mori, X. Jia, T. Takahashi, A. Wernerson, M. Flodström‐Tullberg, A. Tibell, Cell Transplant. 2013, 22, 1137. 23043940 10.3727/096368912X657486

[6] A. M. J. Shapiro, Rev. Diabet. Stud. 2012, 9, 385. 23804275 10.1900/RDS.2012.9.385 PMC3740705

[7] A. M. J. Shapiro, J. R. T. Lakey, E. A. Ryan, G. S. Korbutt, E. Toth, G. L. Warnock, N. M. Kneteman, R. V. Rajotte, N. Engl. J. Med. 2000, 343, 230. 10911004 10.1056/NEJM200007273430401

[8] A. M. Davalli, Y. Ogawa, C. Ricordi, D. W. Scharp, S. Bonner‐weir, G. C. Weir, Transplantation 1995, 59, 817. 7701574

[9] B. Ritz‐Laser, J. Oberholzer, C. Toso, M.‐C. Brulhart, K. Zakrzewska, F. Ris, P. Bucher, P. Morel, J. Philippe, Diabetes 2002, 51, 557. 11872650 10.2337/diabetes.51.3.557

[10] N. R. Barshes, S. Wyllie, J. A. Goss, J. Leukoc. Biol. 2005, 77, 587. 15728243 10.1189/jlb.1104649

[11] V. Delaune, T. Berney, S. Lacotte, C. Toso, Transpl. Int. 2017, 30, 227. 28109023 10.1111/tri.12919

[12] M. D. McCall, C. Toso, E. E. Baetge, A. M. J. Shapiro, Clin. Sci. 2010, 118, 87. 10.1042/CS20090072 19807695

[13] P.‐O. Carlsson, D. Espes, A. Sedigh, A. Rotem, B. Zimerman, H. Grinberg, T. Goldman, U. Barkai, Y. Avni, G. T. Westermark, L. Carlbom, H. Ahlström, O. Eriksson, J. Olerud, O. Korsgren, Am. J. Transplant. 2018, 18, 1735. 29288549 10.1111/ajt.14642 PMC6055594

[14] P. de Vos, in Principles of Tissue Engineering, Elsevier, 2020, pp. 665–679.

[15] J. Magisson, A. Sassi, D. Xhema, A. Kobalyan, P. Gianello, B. Mourer, N. Tran, C. T. Burcez, R. Bou Aoun, S. Sigrist, J. Tissue Eng. 2020, 11, 204173142092481. 10.1177/2041731420924818 PMC7257875 32523669

[16] P. Augsornworawat, K. G. Maxwell, L. Velazco‐Cruz, J. R. Millman, Cell Rep. 2020, 32, 108067. 32846125 10.1016/j.celrep.2020.108067 PMC7491368

[17] O. Veiseh, A. J. Vegas, Adv. Drug Delivery Rev. 2019, 144, 148. 10.1016/j.addr.2019.08.010 PMC6774350 31491445

[18] J. A. Thomson, J. Itskovitz‐Eldor, S. S. Shapiro, M. A. Waknitz, J. J. Swiergiel, V. S. Marshall, J. M. Jones, Science 1998, 282, 1145. 9804556 10.1126/science.282.5391.1145

[19] K. Takahashi, K. Tanabe, M. Ohnuki, M. Narita, T. Ichisaka, K. Tomoda, S. Yamanaka, Cell 2007, 131, 861. 18035408 10.1016/j.cell.2007.11.019

[20] F. W. Pagliuca, D. A. Melton, Development 2013, 140, 2472. 23715541 10.1242/dev.093187 PMC3666377

[21] K. A. D'Amour, A. D. Agulnick, S. Eliazer, O. G. Kelly, E. Kroon, E. E. Baetge, Nat. Biotechnol. 2005, 23, 1534. 16258519 10.1038/nbt1163

[22] K. A. D'Amour, A. G. Bang, S. Eliazer, O. G. Kelly, A. D. Agulnick, N. G. Smart, M. A. Moorman, E. Kroon, M. K. Carpenter, E. E. Baetge, Nat. Biotechnol. 2006, 24, 1392. 17053790 10.1038/nbt1259

[23] E. Kroon, L. A. Martinson, K. Kadoya, A. G. Bang, O. G. Kelly, S. Eliazer, H. Young, M. Richardson, N. G. Smart, J. Cunningham, A. D. Agulnick, K. A. D'Amour, M. K. Carpenter, E. E. Baetge, Nat. Biotechnol. 2008, 26, 443. 18288110 10.1038/nbt1393

[24] A. Rezania, J. E. Bruin, J. Xu, K. Narayan, J. K. Fox, J. J. O'Neil, T. J. Kieffer, Stem Cells 2013, 31, 2432. 23897760 10.1002/stem.1489

[25] J. E. Bruin, A. Rezania, J. Xu, K. Narayan, J. K. Fox, J. J. O'Neil, T. J. Kieffer, Diabetologia 2013, 56, 1987. 23771205 10.1007/s00125-013-2955-4

[26] M. C. Nostro, F. Sarangi, S. Ogawa, A. Holtzinger, B. Corneo, X. Li, S. J. Micallef, I.‐H. Park, C. Basford, M. B. Wheeler, G. Q. Daley, A. G. Elefanty, E. G. Stanley, G. Keller, Development 2011, 138, 861. 21270052 10.1242/dev.055236 PMC3035090

[27] M. Borowiak, R. Maehr, S. Chen, A. E. Chen, W. Tang, J. L. Fox, S. L. Schreiber, D. A. Melton, Cell Stem Cell 2009, 4, 348. 19341624 10.1016/j.stem.2009.01.014 PMC4564293

[28] X. Xu, V. L. Browning, J. S. Odorico, Mech. Dev. 2011, 128, 412. 21855631 10.1016/j.mod.2011.08.001 PMC3225072

[29] M. C. Nostro, F. Sarangi, C. Yang, A. Holland, A. G. Elefanty, E. G. Stanley, D. L. Greiner, G. Keller, Stem Cell Rep. 2015, 4, 591. 10.1016/j.stemcr.2015.02.017 PMC4400642 25843049

[30] F. W. Pagliuca, J. R. Millman, M. Gürtler, M. Segel, A. Van Dervort, J. H. Ryu, Q. P. Peterson, D. Greiner, D. A. Melton, Cell 2014, 159, 428. 25303535 10.1016/j.cell.2014.09.040 PMC4617632

[31] Z. Ghazizadeh, D.‐I. Kao, S. Amin, B. Cook, S. Rao, T. Zhou, T. Zhang, Z. Xiang, R. Kenyon, O. Kaymakcalan, C. Liu, T. Evans, S. Chen, Nat. Commun. 2017, 8, 298. 28824164 10.1038/s41467-017-00129-y PMC5563509

[32] E. Yoshihara, C. O'Connor, E. Gasser, Z. Wei, T. G. Oh, T. W. Tseng, D. Wang, F. Cayabyab, Y. Dai, R. T. Yu, C. Liddle, A. R. Atkins, M. Downes, R. M. Evans, Nature 2020, 586, 606. 32814902 10.1038/s41586-020-2631-z PMC7872080

[33] G. G. Nair, J. S. Liu, H. A. Russ, S. Tran, M. S. Saxton, R. Chen, C. Juang, M. Li, V. Q. Nguyen, S. Giacometti, S. Puri, Y. Xing, Y. Wang, G. L. Szot, J. Oberholzer, A. Bhushan, M. Hebrok, Nat. Cell Biol. 2019, 21, 263. 30710150 10.1038/s41556-018-0271-4 PMC6746427

[34] N. J. Hogrebe, P. Augsornworawat, K. G. Maxwell, L. Velazco‐Cruz, J. R. Millman, Nat. Biotechnol. 2020, 38, 460. 32094658 10.1038/s41587-020-0430-6 PMC7274216

[35] P. U. Mahaddalkar, K. Scheibner, S. Pfluger, M. S. Ansarullah, J. Beckenbauer, M. Irmler, J. Beckers, S. Knöbel, H. Lickert, Nat. Biotechnol. 2020, 38, 1061. 32341565 10.1038/s41587-020-0492-5

[36] O. G. Kelly, M. Y. Chan, L. A. Martinson, K. Kadoya, T. M. Ostertag, K. G. Ross, M. Richardson, M. K. Carpenter, K. A. D'Amour, E. Kroon, M. Moorman, E. E. Baetge, A. G. Bang, Nat. Biotechnol. 2011, 29, 750. 21804561 10.1038/nbt.1931

[37] K. F. Cogger, A. Sinha, F. Sarangi, E. C. McGaugh, D. Saunders, C. Dorrell, S. Mejia‐Guerrero, Y. Aghazadeh, J. L. Rourke, R. A. Screaton, M. Grompe, P. R. Streeter, A. C. Powers, M. Brissova, T. Kislinger, M. C. Nostro, Nat. Commun. 2017, 8, 331. 28835709 10.1038/s41467-017-00561-0 PMC5569081

[38] A. Veres, A. L. Faust, H. L. Bushnell, E. N. Engquist, J. H.‐R. Kenty, G. Harb, Y.‐C. Poh, E. Sintov, M. Gürtler, F. W. Pagliuca, Q. P. Peterson, D. A. Melton, Nature 2019, 569, 368. 31068696 10.1038/s41586-019-1168-5 PMC6903417

[39] X. Li, K. Y. Yang, V. W. Chan, K. T. Leung, X.‐B. Zhang, A. S. Wong, C. C. N. Chong, C. C. Wang, M. Ku, K. O. Lui, Stem Cell Rep. 2020, 15, 1111. 10.1016/j.stemcr.2020.09.009 PMC7663789 33096048

[40] P. Augsornworawat, L. Velazco‐Cruz, J. Song, J. R. Millman, Acta Biomater. 2019, 97, 272. 31446050 10.1016/j.actbio.2019.08.031 PMC6801041

[41] M. Jaramillo, S. Mathew, H. Mamiya, S. K. Goh, I. Banerjee, Tissue Eng., Part A 2015, 21, 14. 24943736 10.1089/ten.tea.2014.0013 PMC4293092

[42] J. Candiello, T. S. P. Grandhi, S. K. Goh, V. Vaidya, M. Lemmon‐Kishi, K. R. Eliato, R. Ros, P. N. Kumta, K. Rege, I. Banerjee, Biomaterials 2018, 177, 27. 29883914 10.1016/j.biomaterials.2018.05.031

[43] D. Talavera‐Adame, O. O. Woolcott, J. Ignatius‐Irudayam, V. Arumugaswami, D. H. Geller, D. C. Dafoe, Diabetologia 2016, 59, 2378. 27567623 10.1007/s00125-016-4078-1 PMC5506104

[44] K. G. Maxwell, P. Augsornworawat, L. Velazco‐Cruz, M. H. Kim, R. Asada, N. J. Hogrebe, S. Morikawa, F. Urano, J. R. Millman, Sci. Transl. Med. 2020, 12, eaax9106. 32321868 10.1126/scitranslmed.aax9106 PMC7233417

[45] M. Yamada, B. Johannesson, I. Sagi, L. C. Burnett, D. H. Kort, R. W. Prosser, D. Paull, M. W. Nestor, M. Freeby, E. Greenberg, R. S. Goland, R. L. Leibel, S. L. Solomon, N. Benvenisty, M. V. Sauer, D. Egli, Nature 2014, 510, 533. 24776804 10.1038/nature13287

[46] J. R. Millman, C. Xie, A. Van Dervort, M. Gürtler, F. W. Pagliuca, D. A. Melton, Nat. Commun. 2016, 7, 11463. 27163171 10.1038/ncomms11463 PMC4866045

[47] B. A. Baghbaderani, X. Tian, B. H. Neo, A. Burkall, T. Dimezzo, G. Sierra, X. Zeng, K. Warren, D. P. Kovarcik, T. Fellner, M. S. Rao, Stem Cell Rep. 2015, 5, 647. 10.1016/j.stemcr.2015.08.015 PMC4624993 26411904

[48] J. R. Millman, F. W. Pagliuca, Diabetes 2017, 66, 1111. 28507211 10.2337/db16-1406

[49] T. Desai, L. D. Shea, Nat. Rev. Drug Discovery 2017, 16, 338. 28008169 10.1038/nrd.2016.232 PMC11286215

[50] A. J. Vegas, O. Veiseh, M. Gürtler, J. R. Millman, F. W. Pagliuca, A. R. Bader, J. C. Doloff, J. Li, M. Chen, K. Olejnik, H. H. Tam, S. Jhunjhunwala, E. Langan, S. Aresta‐Dasilva, S. Gandham, J. J. McGarrigle, M. A. Bochenek, J. Hollister‐Lock, J. Oberholzer, D. L. Greiner, G. C. Weir, D. A. Melton, R. Langer, D. G. Anderson, Nat. Med. 2016.

[51] D. A. Alagpulinsa, J. J. L. Cao, R. K. Driscoll, R. F. Sîrbulescu, M. F. E. Penson, M. Sremac, E. N. Engquist, T. A. Brauns, J. F. Markmann, D. A. Melton, M. C. Poznansky, Am. J. Transplant. 2019, 19, 1930. 30748094 10.1111/ajt.15308

[52] A. A. Stock, V. Manzoli, T. De Toni, M. M. Abreu, Y.‐C. Poh, L. Ye, A. Roose, F. W. Pagliuca, C. Thanos, C. Ricordi, A. A. Tomei, Stem Cell Rep. 2020, 14, 91. 10.1016/j.stemcr.2019.11.004 PMC6962554 31839542

[53] R. L. Youngblood, J. P. Sampson, K. R. Lebioda, L. D. Shea, Acta Biomater. 2019, 96, 111. 31247380 10.1016/j.actbio.2019.06.032 PMC6717676

[54] T. Deuse, X. Hu, A. Gravina, D. Wang, G. Tediashvili, C. De, W. O. Thayer, A. Wahl, J. V. Garcia, H. Reichenspurner, M. M. Davis, L. L. Lanier, S. Schrepfer, Nat. Biotechnol. 2019, 37, 252. 30778232 10.1038/s41587-019-0016-3 PMC6419516

[55] A. S. Lee, C. Tang, M. S. Rao, I. L. Weissman, J. C. Wu, Nat. Med. 2013, 19, 998. 23921754 10.1038/nm.3267 PMC3967018

[56] F. T. Merkle, S. Ghosh, N. Kamitaki, J. Mitchell, Y. Avior, C. Mello, S. Kashin, S. Mekhoubad, D. Ilic, M. Charlton, G. Saphier, R. E. Handsaker, G. Genovese, S. Bar, N. Benvenisty, S. A. McCarroll, K. Eggan, Nature 2017, 545, 229. 28445466 10.1038/nature22312 PMC5427175

[57] M. M. F. Qadir, S. Álvarez‐Cubela, K. Belle, T. Sapir, F. Messaggio, K. B. Johnson, O. Umland, D. Hardin, D. Klein, I. Pérez‐Álvarez, F. Sadiq, O. Alcázar, L. A. Inverardi, C. Ricordi, P. Buchwald, C. A. Fraker, R. L. Pastori, J. Domínguez‐Bendala, Stem Cell Rep. 2019, 12, 611. 10.1016/j.stemcr.2019.01.012 PMC6409439 30773486

[58] Q. Liang, C. Monetti, M. V. Shutova, E. J. Neely, S. Hacibekiroglu, H. Yang, C. Kim, P. Zhang, C. Li, K. Nagy, M. Mileikovsky, I. Gyongy, H.‐K. Sung, A. Nagy, Nature 2018, 563, 701. 30429614 10.1038/s41586-018-0733-7

[59] J. Song, J. R. Millman, Biofabrication 2016, 9, 015002. 27906687 10.1088/1758-5090/9/1/015002 PMC5185469

[60] Z. Zhu, D. Huangfu, Development 2013, 140, 705. 23362344 10.1242/dev.086165 PMC3557771

[61] H. Ohgawara, S. Hirotani, J. Miyazaki, S. Teraoka, Artif. Organs 1998, 22, 788. 9754467 10.1046/j.1525-1594.1998.06185.x

[62] J. Schweicher, C. Nyitray, T. A. Desai, Front. Biosci. 2014, 19, 49. 10.2741/4195 PMC4230297 24389172

[63] B. Memon, E. M. Abdelalim, Cells 2020, 9, 283. 10.3390/cells9020283 PMC7072676 31979403

[64] U. Barkai, A. Rotem, P. de Vos, World J. Transplant. 2016, 6, 69. 27011906 10.5500/wjt.v6.i1.69 PMC4801806

[65] B. L. Gala‐Lopez, A. R. Pepper, P. Dinyari, A. J. Malcolm, T. Kin, L. R. Pawlick, P. A. Senior, A. M. J. Shapiro, CellR4 2016, 4, e2132.

[66] K. Skrzypek, M. G. Nibbelink, L. P. Karbaat, M. Karperien, A. van Apeldoorn, D. Stamatialis, J. Mater. Sci. Mater. Med. 2018, 29, 91. 29938334 10.1007/s10856-018-6102-0 PMC6018599

[67] J. Chen, J. Chen, Y. Cheng, Y. Fu, H. Zhao, M. Tang, H. Zhao, N. Lin, X. Shi, Y. Lei, S. Wang, L. Huang, W. Wu, J. Tan, Stem Cell Res. Ther. 2020, 11, 97. 32127037 10.1186/s13287-020-01610-0 PMC7055095

[68] M. Farina, J. F. Alexander, U. Thekkedath, M. Ferrari, A. Grattoni, Adv. Drug Delivery Rev. 2019, 139, 92. 10.1016/j.addr.2018.04.018 29719210

[69] S. Bose, L. R. Volpatti, D. Thiono, V. Yesilyurt, C. McGladrigan, Y. Tang, A. Facklam, A. Wang, S. Jhunjhunwala, O. Veiseh, J. Hollister‐Lock, C. Bhattacharya, G. C. Weir, D. L. Greiner, R. Langer, D. G. Anderson, Nat. Biomed. Eng. 2020, 4, 814. 32231313 10.1038/s41551-020-0538-5 PMC8051527

[70] Y. Evron, C. K. Colton, B. Ludwig, G. C. Weir, B. Zimermann, S. Maimon, T. Neufeld, N. Shalev, T. Goldman, A. Leon, K. Yavriyants, N. Shabtay, T. Rozenshtein, D. Azarov, A. R. DiIenno, A. Steffen, P. de Vos, S. R. Bornstein, U. Barkai, A. Rotem, Sci. Rep. 2018, 8, 6508. 29695723 10.1038/s41598-018-23862-w PMC5917036

[71] C. Ricordi, D. W. R. Gray, B. J. Hering, D. B. Kaufman, G. L. Warnock, N. M. Kneteman, S. P. Lake, N. J. M. London, C. Socci, R. Alejandro, Y. Zeng, D. W. Scharp, G. Viviani, L. Falqui, A. Tzakis, R. G. Bretzel, K. Federlin, G. Pozza, R. F. L. James, R. V. Rajotte, V. Di Carlo, P. J. Morris, D. E. R. Sutherland, T. E. Starzl, D. H. Mintz, P. E. Lacy, Acta Diabetol. Lat. 1990, 27, 185. 2075782 10.1007/BF02581331

[72] P. de Vos, P. Marchetti, Trends Mol. Med. 2002, 8, 363. 12127717 10.1016/s1471-4914(02)02381-x

[73] D. C. Chow, L. A. Wenning, W. M. Miller, E. T. Papoutsakis, Biophys. J. 2001, 81, 675. 11463616 10.1016/S0006-3495(01)75732-3 PMC1301544

[74] M. Radisic, J. Malda, E. Epping, W. Geng, R. Langer, G. Vunjak‐Novakovic, Biotechnol. Bioeng. 2006, 93, 332. 16270298 10.1002/bit.20722

[75] D. W. Scharp, P. Marchetti, Adv. Drug Delivery Rev. 2014, 67–68, 35. 10.1016/j.addr.2013.07.018 23916992

[76] D. T. Luttikhuizen, M. C. Harmsen, M. J. A. Van Luyn, Tissue Eng. 2006, 12, 1955. 16889525 10.1089/ten.2006.12.1955

[77] W. K. Ward, J. Diabetes Sci. Technol. 2008, 2, 768. 19885259 10.1177/193229680800200504 PMC2769792

[78] B. N. Kharbikar, G. S. Chendke, T. A. Desai, Adv. Drug Delivery Rev. 2021. 10.1016/j.addr.2021.01.011 PMC8217111 33484736

[79] D. T. Bowers, W. Song, L.‐H. Wang, M. Ma, Acta Biomater. 2019, 95, 131. 31128322 10.1016/j.actbio.2019.05.051 PMC6824722

[80] N. Trivedi, G. M. Steil, C. K. Colton, S. Bonner‐Weir, G. C. Weir, Cell Transplant. 2000, 9, 115. 10784073 10.1177/096368970000900114

[81] A. E. Vlahos, I. Talior‐Volodarsky, S. M. Kinney, M. V. Sefton, Biomaterials 2021, 269, 120499. 33168223 10.1016/j.biomaterials.2020.120499

[82] R. Krishnan, M. Alexander, L. Robles, C. E. Foster, J. R. T. Lakey, Rev. Diabet. Stud. 2014, 11, 84. 25148368 10.1900/RDS.2014.11.84 PMC4295801

[83] D. M. Berman, J. J. O'Neil, L. C. K. Coffey, P. C. J. Chaffanjon, N. M. Kenyon, P. Ruiz, A. Pileggi, C. Ricordi, N. S. Kenyon, Am. J. Transplant. 2008, 9, 91. 10.1111/j.1600-6143.2008.02489.x PMC4441095 19133931

[84] A. A. Tomei, C. Villa, C. Ricordi, ExpertOpin. Biol. Ther. 2015, 15, 1321. 10.1517/14712598.2015.1055242 PMC6329003 26156291

[85] J. Svensson, J. Lau, M. Sandberg, P.‐O. Carlsson, Cell Transplant. 2011, 20, 783. 21054943 10.3727/096368910X536527

[86] G. P. Duffy, S. T. Robinson, R. O'Connor, R. Wylie, C. Mauerhofer, G. Bellavia, S. Straino, F. Cianfarani, K. Mendez, R. Beatty, R. Levey, J. O'Sullivan, L. McDonough, H. Kelly, E. T. Roche, E. B. Dolan, Adv. Healthcare Mater. 2020, 9, 2070035. 10.1002/adhm.202000305 32339411

[87] P. Augsornworawat, K. G. Maxwell, L. Velazco‐Cruz, J. R. Millman, Cell Rep. 2020, 32, 108067. 32846125 10.1016/j.celrep.2020.108067 PMC7491368

[88] S. Hrvatin, C. W. O'Donnell, F. Deng, J. R. Millman, F. W. Pagliuca, P. DiIorio, A. Rezania, D. K. Gifford, D. A. Melton, Proc. Natl. Acad. Sci 2014, 111, 3038. 24516164 10.1073/pnas.1400709111 PMC3939927

[89] M. Figliuzzi, R. Cornolti, N. Perico, C. Rota, M. Morigi, G. Remuzzi, A. Remuzzi, A. Benigni, Transplant. Proc. 2009, 41, 1797. 19545731 10.1016/j.transproceed.2008.11.015

[90] C. E. Nyitray, M. G. Chavez, T. A. Desai, Tissue Eng., Part A 2014, 20, 1888. 24433489 10.1089/ten.tea.2013.0692 PMC4085995

[91] J. C. Stendahl, D. B. Kaufman, S. I. Stupp, Cell Transplant. 2009, 18, 1. 19476204 10.3727/096368909788237195 PMC2724969

[92] A. Llacua, B. J. de Haan, S. A. Smink, P. de Vos, J. Biomed. Mater. Res. Part A 2016, 104, 1788. 10.1002/jbm.a.35706 26990360

[93] L. A. Llacua, B. J. de Haan, P. de Vos, J. Tissue Eng. Regener. Med. 2018, 12, 460. 10.1002/term.2472 28508555

[94] S. V. Bhujbal, B. de Haan, S. P. Niclou, P. de Vos, Sci. Rep. 2015, 4, 6856. 10.1038/srep06856 PMC4215319 25358640

[95] A. M. J. Shapiro, C. Ricordi, B. J. Hering, H. Auchincloss, R. Lindblad, R. P. Robertson, A. Secchi, M. D. Brendel, T. Berney, D. C. Brennan, E. Cagliero, R. Alejandro, E. A. Ryan, B. DiMercurio, P. Morel, K. S. Polonsky, J.‐A. Reems, R. G. Bretzel, F. Bertuzzi, T. Froud, R. Kandaswamy, D. E. R. Sutherland, G. Eisenbarth, M. Segal, J. Preiksaitis, G. S. Korbutt, F. B. Barton, L. Viviano, V. Seyfert‐Margolis, J. Bluestone, J. R. T. Lakey, N. Engl. J. Med. 2006, 355, 1318. 17005949 10.1056/NEJMoa061267

[96] U. Barkai, G. C. Weir, C. K. Colton, B. Ludwig, S. R. Bornstein, M. D. Brendel, T. Neufeld, C. Bremer, A. Leon, Y. Evron, K. Yavriyants, D. Azarov, B. Zimermann, S. Maimon, N. Shabtay, M. Balyura, T. Rozenshtein, P. Vardi, K. Bloch, P. De Vos, A. Rotem, Cell Transplant. 2013, 22, 1463. 23043896 10.3727/096368912X657341

[97] R. Lehmann, M. Weber, P. Berthold, R. Zullig, T. Pfammatter, W. Moritz, K. Madler, M. Donath, P. Ambuhl, N. Demartines, P.‐A. Clavien, G. A. Spinas, Am. J. Transplant. 2004, 4, 1117. 15196070 10.1111/j.1600-6143.2004.00468.x

[98] J. A. Emamaullee, A. M. J. Shapiro, Cell Transplant. 2007, 16, 1. 17436849

[99] K. E. Dionne, C. K. Colton, M. Lyarmush, Diabetes 1993, 42, 12. 8420809 10.2337/diab.42.1.12

[100] N. Wang, S. A. Khan, N. R. Prabhakar, J. Nanduri, Exp. Physiol. 2013, 98, 1376. 23709585 10.1113/expphysiol.2013.072454 PMC3756548

[101] K. K. Papas, R. C. Long, I. Constantinidis, A. Sambanis, Biochim. Biophys. Acta – Gen. Subj. 1996, 1291, 163. 10.1016/0304-4165(96)00062-1 8898878

[102] A. C. Kelly, K. E. Smith, W. G. Purvis, C. G. Min, C. S. Weber, A. M. Cooksey, C. Hasilo, S. Paraskevas, T. M. Suszynski, B. P. Weegman, M. J. Anderson, L. E. Camacho, R. C. Harland, T. Loudovaris, J. Jandova, D.i. S. Molano, N. D. Price, I. G. Georgiev, W. E. Scott, D. M. D. Manas, J. A. M. Shaw, D. O'Gorman, T. Kin, F. M. McCarthy, G. L. Szot, A. M. Posselt, P. G. Stock, T. Karatzas, A. M. J. Shapiro, R. M. Lynch, S. W. Limesand, K. K. Papas, Transplantation 2019, 103, 160. 30095738 10.1097/TP.0000000000002400 PMC6371803

[103] S. Pitchumoni, M. R. Garfinkel, E. D. Littman, E. C. Opara, Metabolism 1998, 47, 809. 9667226 10.1016/s0026-0495(98)90117-2

[104] Y. Lu, X. Jin, Y. Chen, S. Li, Y. Yuan, G. Mai, B. Tian, D. Long, J. Zhang, L. Zeng, Y. Li, J. Cheng, Cell Biochem. Funct. 2010, 28, 637. 21061411 10.1002/cbf.1701

[105] H. Komatsu, C. Cook, C.‐H. Wang, L. Medrano, H. Lin, F. Kandeel, Y.‐C. Tai, Y. Mullen, PLoS One 2017, 12, e0183780. 28832685 10.1371/journal.pone.0183780 PMC5568442

[106] R. Lehmann, R. A. Zuellig, P. Kugelmeier, P. B. Baenninger, W. Moritz, A. Perren, P. A. Clavien, M. Weber, G. A. Spinas, Diabetes 2007, 56, 594. 17327426 10.2337/db06-0779

[107] M. Garcia‐Contreras, A. Tamayo‐Garcia, K. L. Pappan, G. A. Michelotti, C. L. Stabler, C. Ricordi, P. Buchwald, J. Proteome Res. 2017, 16, 2294. 28452488 10.1021/acs.jproteome.7b00160 PMC5557342

[108] G. Faleo, H. A. Russ, S. Wisel, A. V. Parent, V. Nguyen, G. G. Nair, J. E. Freise, K. E. Villanueva, G. L. Szot, M. Hebrok, Q. Tang, Stem Cell Rep. 2017, 9, 807. 10.1016/j.stemcr.2017.07.012 PMC5599226 28803916

[109] C. K. Colton, Adv. Drug Delivery Rev. 2014, 67–68, 93. 10.1016/j.addr.2014.02.007 24582600

[110] U. Utzinger, B. Baggett, J. A. Weiss, J. B. Hoying, L. T. Edgar, Angiogenesis 2015, 18, 219. 25795217 10.1007/s10456-015-9461-x PMC4782613

[111] M. W. Laschke, M. D. Menger, Biotechnol. Adv. 2016, 34, 112. 26674312 10.1016/j.biotechadv.2015.12.004

[112] A. K. Sörenby, M. Kumagai‐Braesch, A. Sharma, K. R. Hultenby, A. M. Wernerson, A. B. Tibell, Transplantation 2008, 86, 364. 18645504 10.1097/TP.0b013e31817efc78

[113] A. R. Pepper, B. Gala‐Lopez, R. Pawlick, S. Merani, T. Kin, A. M. J. Shapiro, Nat. Biotechnol. 2015, 33, 518. 25893782 10.1038/nbt.3211

[114] P. O. Carlsson, P. Liss, A. Andersson, L. Jansson, Diabetes 1998, 47, 1027. 9648824 10.2337/diabetes.47.7.1027

[115] J. Rouwkema, N. C. Rivron, C. A. van Blitterswijk, Trends Biotechnol. 2008, 26, 434. 18585808 10.1016/j.tibtech.2008.04.009

[116] R. Gianni‐Barrera, N. Di Maggio, L. Melly, M. G. Burger, E. Mujagic, L. Gürke, D. J. Schaefer, A. Banfi, Stem Cells Transl. Med. 2020, 9, 433. 31922362 10.1002/sctm.19-0319 PMC7103618

[117] P. Carmeliet, Nat. Med. 2000, 6, 389. 10742145 10.1038/74651

[118] M. Kastellorizios, F. Papadimitrakopoulos, D. J. Burgess, J. Controlled Release 2015, 214, 103. 10.1016/j.jconrel.2015.07.021 26216396

[119] M. Kastellorizios, F. Papadimitrakopoulos, D. J. Burgess, J. Controlled Release 2015, 202, 101. 10.1016/j.jconrel.2015.01.038 25645376

[120] N. M. Luan, H. Iwata, Am. J. Transplant. 2014, 14, 1533. 24909185 10.1111/ajt.12739

[121] J. H. Brauker, V. E. Carr‐Brendel, L. A. Martinson, J. Crudele, W. D. Johnston, R. C. Johnson, J. Biomed. Mater. Res. 1995, 29, 1517. 8600142 10.1002/jbm.820291208

[122] R. B. Elliott, L. Escobar, R. Calafiore, G. Basta, O. Garkavenko, A. Vasconcellos, C. Bambra, Transplant. Proc. 2005, 37, 466. 15808678 10.1016/j.transproceed.2004.12.198

[123] A. R. Pepper, R. Pawlick, B. Gala‐Lopez, A. MacGillivary, D. M. Mazzuca, D. J. G. White, P. M. Toleikis, A. M. J. Shapiro, Transplantation 2015, 99, 2294. 26308506 10.1097/TP.0000000000000864 PMC4623852

[124] N. Khosravi, A. Maeda, R. S. DaCosta, J. E. Davies, Commun. Biol. 2018, 1, 72. 30271953 10.1038/s42003-018-0074-y PMC6123776

[125] K. K. Papas, H. De Leon, T. M. Suszynski, R. C. Johnson, Adv. Drug Delivery Rev. 2019, 139, 139. 10.1016/j.addr.2019.05.002 31077781

[126] A. Nandi, L. J. Yan, C. K. Jana, N. Das, Oxid. Med. Cell. Longev. 2019, 2019, 1. 10.1155/2019/9613090 PMC6885225 31827713

[127] S. H. Oh, C. L. Ward, A. Atala, J. J. Yoo, B. S. Harrison, Biomaterials 2009, 30, 757. 19019425 10.1016/j.biomaterials.2008.09.065

[128] J. Wang, Y. Zhu, H. K. Bawa, G. Ng, Y. Wu, M. Libera, H. C. van der Mei, H. J. Busscher, X. Yu, ACS Appl. Mater. Interfaces 2011, 3, 67. 21155527 10.1021/am100862h

[129] A. Motealleh, N. S. Kehr, J. Mater. Chem. B 2020, 8, 4195. 32393933 10.1039/d0tb00885k

[130] M. M. Coronel, R. Geusz, C. L. Stabler, Biomaterials 2017, 129, 139. 28342320 10.1016/j.biomaterials.2017.03.018 PMC5497707

[131] M. M. Coronel, J.‐P. Liang, Y. Li, C. L. Stabler, Biomaterials 2019, 210, 1. 31029812 10.1016/j.biomaterials.2019.04.017 PMC6527135

[132] Y. Guan, N. Gao, H. Niu, Y. Dang, J. Guan, J. Controlled Release 2021, 331, 376. 10.1016/j.jconrel.2021.01.034 PMC8007231 33508351

[133] D. R. Spahn, Crit. Care 2018, 22, 46. 29471841 10.1186/s13054-018-1949-5 PMC5824566

[134] J. G. Riess, Artif. Cells, Blood Substitutes, Biotechnol. 2005, 33, 47. 10.1081/bio-200046659 15768565

[135] J. G. Riess, Chem. Rev. 2001, 101, 2797. 11749396 10.1021/cr970143c

[136] S. A. Hosgood, M. L. Nicholson, Transplantation 2010, 89, 1169. 20393403 10.1097/TP.0b013e3181da6064

[137] H. Brandhorst, S. Asif, K. Andersson, B. Theisinger, H. H. Andersson, M. Felldin, A. Foss, K. Salmela, A. Tibell, G. Tufveson, O. Korsgren, D. Brandhorst, Transplantation 2010, 89, 155. 20098277 10.1097/TP.0b013e3181c9266c

[138] T. Mitsuno, H. Ohyanagi, R. Naito, Ann. Surg. 1982, 195, 60. 7034658 10.1097/00000658-198201001-00010 PMC1352405

[139] E. Maillard, M. T. Juszczak, A. Langlois, C. Kleiss, M. C. Sencier, W. Bietiger, M. Sanchez‐Dominguez, M. P. Krafft, P. R. V. Johnson, M. Pinget, S. Sigrist, Cell Transplant. 2012, 21, 657. 21944582 10.3727/096368911X593136

[140] S. Lee, H. Park, Y. Yang, E. Lee, J. Kim, G. Khang, K. Yoon, J. Tissue Eng. Regener. Med. 2018, 12, e2110. 10.1002/term.2643 29330944

[141] T. E. L. Douglas, M. Pilarek, I. Kalaszczyńska, I. Senderek, A. Skwarczyńska, V. M. J. I. Cuijpers, Z. Modrzejewska, M. Lewandowska‐Szumieł, P. Dubruel, Mater. Lett. 2014, 128, 79.

[142] E. Maillard, M. T. Juszczak, A. Clark, S. J. Hughes, D. R. W. Gray, P. R. V. Johnson, Biomaterials 2011, 32, 9282. 21899883 10.1016/j.biomaterials.2011.08.044

[143] H. Bergert, K.‐P. Knoch, R. Meisterfeld, M. Jäger, J. Ouwendijk, S. Kersting, H. D. Saeger, M. Solimena, Cell Transplant. 2005, 14, 441. 16285252 10.3727/000000005783982873

[144] A. S. Johnson, E. O'Sullivan, L. N. D'Aoust, A. Omer, S. Bonner‐Weir, R. J. Fisher, G. C. Weir, C. K. Colton, Tissue Eng., Part C 2011, 17, 435. 10.1089/ten.tec.2009.0510 PMC3065730 21067465

[145] A. S. Johnson, R. J. Fisher, G. C. Weir, C. K. Colton, Chem. Eng. Sci. 2009, 64, 4470.

[146] A. S. Benitez Cardenas, P. P. Samuel, J. S. Olson, Shock 2019, 52, 28. 29112633 10.1097/SHK.0000000000001053 PMC5936680

[147] A. S. Gupta, Shock 2019, 52, 70.31513123

[148] F. L.e Pape, L. Cosnuau‐Kemmat, G. Richard, F. Dubrana, C. Férec, F. Zal, E. Leize, P. Delépine, Artif. Organs 2017, 41, 359. 28326561 10.1111/aor.12892

[149] A. Rodriguez‐Brotons, W. Bietiger, C. Peronet, A. Langlois, J. Magisson, C. Mura, C. Sookhareea, V. Polard, N. Jeandidier, F. Zal, M. Pinget, S. Sigrist, E. Maillard, Tissue Eng., Part A 2016, 22, 1327. 27796164 10.1089/ten.TEA.2016.0064

[150] M. Rousselot, E. Delpy, C. Drieu La Rochelle, V. Lagente, R. Pirow, J.‐F. Rees, A. Hagege, D. Le Guen, S. Hourdez, F. Zal, Biotechnol. J. 2006, 1, 333. 16897713 10.1002/biot.200500049

[151] H. Komatsu, C. A. Cook, N. Gonzalez, L. Medrano, M. Salgado, F. Sui, J. Li, F. Kandeel, Y. Mullen, Y.‐C. Tai, Biofabrication 2018, 11, 015011. 30524058 10.1088/1758-5090/aaf2f0 PMC9851375

[152] B. Ludwig, A. Rotem, J. Schmid, G. C. Weir, C. K. Colton, M. D. Brendel, T. Neufeld, N. L. Block, K. Yavriyants, A. Steffen, S. Ludwig, T. Chavakis, A. Reichel, D. Azarov, B. Zimermann, S. Maimon, M. Balyura, T. Rozenshtein, N. Shabtay, P. Vardi, K. Bloch, P. De Vos, A. V. Schally, S. R. Bornstein, U. Barkai, Proc. Natl. Acad. Sci 2012, 109, 5022. 22393012 10.1073/pnas.1201868109 PMC3324017

[153] D. Kang, H. Komatsu, H. Lin, C. A. Cook, Y.‐C. Tai, Y. Mullen, F. R. Kandeel, In 2016 IEEE 11th Annual International Conference on Nano/Micro Engineered and Molecular Systems (NEMS), IEEE, 2016, pp. 499–503.

[154] B. Ludwig, B. Zimerman, A. Steffen, K. Yavriants, D. Azarov, A. Reichel, P. Vardi, T. German, N. Shabtay, A. Rotem, Y. Evron, T. Neufeld, S. Mimon, S. Ludwig, M. D. Brendel, S. R. Bornstein, U. Barkai, Horm. Metab. Res. 2010, 42, 918. 21031332 10.1055/s-0030-1267916

[155] T. Neufeld, B. Ludwig, U. Barkai, G. C. Weir, C. K. Colton, Y. Evron, M. Balyura, K. Yavriyants, B. Zimermann, D. Azarov, S. Maimon, N. Shabtay, T. Rozenshtein, D. Lorber, A. Steffen, U. Willenz, K. Bloch, P. Vardi, R. Taube, P. de Vos, E. C. Lewis, S. R. Bornstein, A. Rotem, PLoS One 2013, 8, e70150. 23936385 10.1371/journal.pone.0070150 PMC3731363

[156] B. Ludwig, S. Ludwig, A. Steffen, Y. Knauf, B. Zimerman, S. Heinke, S. Lehmann, U. Schubert, J. Schmid, M. Bleyer, U. Schönmann, C. K. Colton, E. Bonifacio, M. Solimena, A. Reichel, A. V. Schally, A. Rotem, U. Barkai, H. Grinberg‐Rashi, F.‐J. Kaup, Y. Avni, P. Jones, S. R. Bornstein, Proc. Natl. Acad. Sci 2017, 114, 11745. 29078330 10.1073/pnas.1708420114 PMC5676906

[157] R. Sudds, Bio‐artificial pancreas on track for Type‐I diabetes cure, https://patch.com/massachusetts/boston/bio‐artificial‐pancreas‐track‐type‐i‐diabetes‐cure (accessed March 2020), p. 2021.

[158] Beta O2 Technologies Ltd. , Clinical trials, https://beta‐o2.com/clinical‐trials/ (accessed March 2021).

[159] H. Wu, E. S. Avgoustiniatos, L. Swette, S. Bonner‐Weir, G. C. Weir, C. K. Colton, Ann. N. Y. Acad. Sci. 1999, 875, 105. 10415561 10.1111/j.1749-6632.1999.tb08497.x

[160] Procyon Technologies LLC , Procyon Technologies LLC and Novo Nordisk A/S to collaborate on the development of a stem‐cell based therapy for Type 1 diabetes, https://www.prnewswire.com/news‐releases/procyon‐technologies‐llc‐and‐novo‐nordisk‐as‐to‐collaborate‐on‐the‐development‐of‐a‐stem‐cell‐based‐therapy‐for‐type‐1‐diabetes‐301188131.html (accessed: March 2021).

[161] Giner Life Sciences , Applications for Giner Life Sciences, https://www.ginerinc.com/life‐sciences‐applications (accessed: March 2021).

[162] L. A. Tempelman, S. G. Stone, K. K. Papas, US Patent US10231817B2, 2019.

[163] E. Mariani, G. Lisignoli, R. M. Borzì, L. Pulsatelli, Int. J. Mol. Sci. 2019, 20, 636. 10.3390/ijms20030636 PMC6386828 30717232

[164] B. F. Matlaga, L. P. Yasenchak, T. N. Salthouse, J. Biomed. Mater. Res. 1976, 10, 391. 1270456 10.1002/jbm.820100308

[165] T. N. Salthouse, J. Biomed. Mater. Res. 1984, 18, 395. 6234318 10.1002/jbm.820180407

[166] O. Veiseh, J. C. Doloff, M. Ma, A. J. Vegas, H. H. Tam, A. R. Bader, J. Li, E. Langan, J. Wyckoff, W. S. Loo, S. Jhunjhunwala, A. Chiu, S. Siebert, K. Tang, J. Hollister‐Lock, S. Aresta‐Dasilva, M. Bochenek, J. Mendoza‐Elias, Y. Wang, M. Qi, D. M. Lavin, M. Chen, N. Dholakia, R. Thakrar, I. Lacík, G. C. Weir, J. Oberholzer, D. L. Greiner, R. Langer, D. G. Anderson, Nat. Mater. 2015, 14, 643. 25985456 10.1038/nmat4290 PMC4477281

[167] W. K. Ward, E. P. Slobodzian, K. L. Tiekotter, M. D. Wood, Biomaterials 2002, 23, 4185. 12194521 10.1016/s0142-9612(02)00160-6

[168] L. R. Madden, D. J. Mortisen, E. M. Sussman, S. K. Dupras, J. A. Fugate, J. L. Cuy, K. D. Hauch, M. A. Laflamme, C. E. Murry, B. D. Ratner, Proc. Natl. Acad. Sci 2010, 107, 15211. 20696917 10.1073/pnas.1006442107 PMC2930533

[169] C. E. Witherel, K. Sao, B. K. Brisson, B. Han, S. W. Volk, R. J. Petrie, H. Lin, K. L. Spiller, Biomaterials 2021, 269, 120667. 33450585 10.1016/j.biomaterials.2021.120667 PMC7870567

[170] B. N. Brown, R. Londono, S. Tottey, L. Zhang, K. A. Kukla, M. T. Wolf, K. A. Daly, J. E. Reing, S. F. Badylak, Acta Biomater. 2012, 8, 978. 22166681 10.1016/j.actbio.2011.11.031 PMC4325370

[171] E. M. Sussman, M. C. Halpin, J. Muster, R. T. Moon, B. D. Ratner, Ann. Biomed. Eng. 2014, 42, 1508. 24248559 10.1007/s10439-013-0933-0

[172] P. E. Scopelliti, A. Borgonovo, M. Indrieri, L. Giorgetti, G. Bongiorno, R. Carbone, A. Podestà, P. Milani, PLoS One 2010, 5, e11862. 20686681 10.1371/journal.pone.0011862 PMC2912332

[173] P. Roach, D. Eglin, K. Rohde, C. C. Perry, J. Mater. Sci. Mater. Med. 2007, 18, 1263. 17443395 10.1007/s10856-006-0064-3

[174] M. P. Vincent, S. Bobbala, N. B. Karabin, M. Frey, Y. Liu, J. O. Navidzadeh, T. Stack, E. A. Scott, Nat. Commun. 2021, 12, 648. 33510170 10.1038/s41467-020-20886-7 PMC7844416

[175] M. Hulander, Lundgren, Berglin, Ohrlander, Lausmaa, Elwing, Int. J. Nanomed. 2011, 2653. 10.2147/IJN.S24578 PMC3218579 22114496

[176] E. B. Dolan, C. E. Varela, K. Mendez, W. Whyte, R. E. Levey, S. T. Robinson, E. Maye, J. O'Dwyer, R. Beatty, A. Rothman, Y. Fan, J. Hochstein, S. E. Rothenbucher, R. Wylie, J. R. Starr, M. Monaghan, P. Dockery, G. P. Duffy, E. T. Roche, Sci. Robot. 2019, 4, eaax7043. 33137787 10.1126/scirobotics.aax7043

[177] M. Brissova, A. Shostak, C. L. Fligner, F. L. Revetta, M. K. Washington, A. C. Powers, R. L. Hull, J. Histochem. Cytochem. 2015, 63, 637. 26216139 10.1369/0022155415573324 PMC4530394

[178] A. J. Vegas, O. Veiseh, J. C. Doloff, M. Ma, H. H. Tam, K. Bratlie, J. Li, A. R. Bader, E. Langan, K. Olejnik, P. Fenton, J. W. Kang, J. Hollister‐Locke, M. A. Bochenek, A. Chiu, S. Siebert, K. Tang, S. Jhunjhunwala, S. Aresta‐Dasilva, N. Dholakia, R. Thakrar, T. Vietti, M. Chen, J. Cohen, K. Siniakowicz, M. Qi, J. McGarrigle, A. C. Graham, S. Lyle, D. M. Harlan, D. L. Greiner, J. Oberholzer, G. C. Weir, R. Langer, D. G. Anderson, Nat. Biotechnol. 2016, 34, 345. 26807527 10.1038/nbt.3462 PMC4904301

[179] S. Farah, J. C. Doloff, P. Müller, A. Sadraei, H. J. Han, K. Olafson, K. Vyas, H. H. Tam, J. Hollister‐Lock, P. S. Kowalski, M. Griffin, A. Meng, M. McAvoy, A. C. Graham, J. McGarrigle, J. Oberholzer, G. C. Weir, D. L. Greiner, R. Langer, D. G. Anderson, Nat. Mater. 2019, 18, 892. 31235902 10.1038/s41563-019-0377-5 PMC7184801

[180] P. X. Ma, Adv. Drug Delivery Rev. 2008, 60, 184. 10.1016/j.addr.2007.08.041 PMC2271038 18045729

[181] M. Sandor, D. Singh, R. P. Silverman, H. Xu, P. G. De Deyne, Eplasty 2014, 14, 52. PMC3914385 24570768

[182] S. Mukherjee, S. Darzi, K. Paul, F. L. Cousins, J. A. Werkmeister, C. E. Gargett, Front. Pharmacol. 2020, 11, 1. 32265721 10.3389/fphar.2020.00353 PMC7107042

[183] M. T. Wolf, C. L. Dearth, C. A. Ranallo, S. T. LoPresti, L. E. Carey, K. A. Daly, B. N. Brown, S. F. Badylak, Biomaterials 2014, 35, 6838. 24856104 10.1016/j.biomaterials.2014.04.115 PMC4347831

[184] J. C. Doloff, O. Veiseh, A. J. Vegas, H. H. Tam, M. Ma, J. Li, A. Bader, A. Chiu, A. Sadraei, S. Aresta‐dasilva, M. Griffin, S. Jhunjhunwala, S. Siebert, K. Tang, M. Chen, E. Langan, R. Thakrar, M. Qi, J. Oberholzer, D. L. Greiner, Nat. Mater. 2017, 16, 671. 28319612 10.1038/nmat4866 PMC5445003

[185] N. Ricard, J. Zhang, Z. W. Zhuang, M. Simons, Cells 2019, 9, 38. 10.3390/cells9010038 PMC7017123 31877781

[186] A. Gamble, A. R. Pepper, A. Bruni, A. M. J. Shapiro, Islets 2018, 10, 80. 29394145 10.1080/19382014.2018.1428511 PMC5895174

[187] W. Whyte, E. T. Roche, C. E. Varela, K. Mendez, S. Islam, H. O'Neill, F. Weafer, R. N. Shirazi, J. C. Weaver, N. V. Vasilyev, P. E. McHugh, B. Murphy, G. P. Duffy, C. J. Walsh, D. J. Mooney, Nat. Biomed. Eng. 2018, 2, 416. 31011199 10.1038/s41551-018-0247-5

[188] J. W. Pickering, M. P. Than, L. Cullen, S. Aldous, E. ter Avest, R. Body, E. W. Carlton, P. Collinson, A. M. Dupuy, U. Ekelund, K. M. Eggers, C. M. Florkowski, Y. Freund, P. George, S. Goodacre, J. H. Greenslade, A. S. Jaffe, S. J. Lord, A. Mokhtari, C. Mueller, A. Munro, S. Mustapha, W. Parsonage, W. F. Peacock, C. Pemberton, A. M. Richards, J. Sanchis, L. P. Staub, R. Troughton, R. Twerenbold, K. Wildi, J. Young, Ann. Intern. Med. 2017, 166, 715. 28418520 10.7326/M16-2562

[189] N. Arroyo‐Currás, J. Somerson, P. A. Vieira, K. L. Ploense, T. E. Kippin, K. W. Plaxco, Proc. Natl. Acad. Sci 2017, 114, 645. 28069939 10.1073/pnas.1613458114 PMC5278471

[190] S. Puhr, P. Calhoun, J. B. Welsh, T. C. Walker, Diabetes Technol. Ther. 2018, 20, 557. 30036082 10.1089/dia.2018.0134

[191] R. Hovorka, Diabet. Med. 2006, 23, 1. 10.1111/j.1464-5491.2005.01672.x 16409558

[192] GlySens , Long‐term CGM is the key to improved diabetes health, http://glysens.com/?page_id=40 (accessed: March 2021).

[193] D. A. Gough, L. S. Kumosa, T. L. Routh, J. T. Lin, J. Y. Lucisano, Sci. Transl. Med. 2010, 2, 42ra53. 10.1126/scitranslmed.3001148 PMC4528300 20668297

[194] J. Y. Lucisano, T. L. Routh, J. T. Lin, D. A. Gough, IEEE Trans. Biomed. Eng. 2017, 64, 1982. 27775510 10.1109/TBME.2016.2619333 PMC5561509

[195] C. Boss, U. De Marchi, A. Hermant, M. Conrad, F. Sizzano, A. Palini, A. Wiederkehr, N. Bouche, Adv. Healthc. Mater 2017, 6, 1600869. 10.1002/adhm.201600869 27995762

[196] A. Graf, S. A. McAuley, C. Sims, J. Ulloa, A. J. Jenkins, G. Voskanyan, D. N. O'Neal, J. Diabetes Sci. Technol. 2017, 11, 308. 28264192 10.1177/1932296816682762 PMC5478040

